# Multifunctional Iron Oxide Magnetic Nanoparticles for Biomedical Applications: A Review

**DOI:** 10.3390/ma15020503

**Published:** 2022-01-10

**Authors:** Hung-Vu Tran, Nhat M. Ngo, Riddhiman Medhi, Pannaree Srinoi, Tingting Liu, Supparesk Rittikulsittichai, T. Randall Lee

**Affiliations:** 1 Department of Chemistry and the Texas Center for Superconductivity, University of Houston, 4800 Calhoun Road, Houston, TX 77204-5003, USA; hvtran2@central.uh.edu (H.-V.T.); nngominh@uh.edu (N.M.N.); riddhi.m.108@gmail.com (R.M.); sdzbltt@gmail.com (T.L.); yai_xl@hotmail.com (S.R.); 2 Department of Chemistry and Centre of Excellence for Innovation in Chemistry, Faculty of Science, Kasetsart University, Bangkok 10900, Thailand; pannaree.sr@ku.ac.th

**Keywords:** magnetic nanoparticles, Fe_3_O_4_, biomedical applications, surface functionalization, organic dyes, biomolecules, stimuli-responsive polymers, quantum dots, metal nanoparticles, magnetic resonance imaging, fluorescence imaging, photodynamic therapy, drug delivery, diagnosis, bacterial detection, cancer treatment, antibacterial, tumor targeting, photothermal ablation

## Abstract

Due to their good magnetic properties, excellent biocompatibility, and low price, magnetic iron oxide nanoparticles (IONPs) are the most commonly used magnetic nanomaterials and have been extensively explored in biomedical applications. Although magnetic IONPs can be used for a variety of applications in biomedicine, most practical applications require IONP-based platforms that can perform several tasks in parallel. Thus, appropriate engineering and integration of magnetic IONPs with different classes of organic and inorganic materials can produce multifunctional nanoplatforms that can perform several functions simultaneously, allowing their application in a broad spectrum of biomedical fields. This review article summarizes the fabrication of current composite nanoplatforms based on integration of magnetic IONPs with organic dyes, biomolecules (e.g., lipids, DNAs, aptamers, and antibodies), quantum dots, noble metal NPs, and stimuli-responsive polymers. We also highlight the recent technological advances achieved from such integrated multifunctional platforms and their potential use in biomedical applications, including dual-mode imaging for biomolecule detection, targeted drug delivery, photodynamic therapy, chemotherapy, and magnetic hyperthermia therapy.

## 1. Introduction

**Figure 1:**
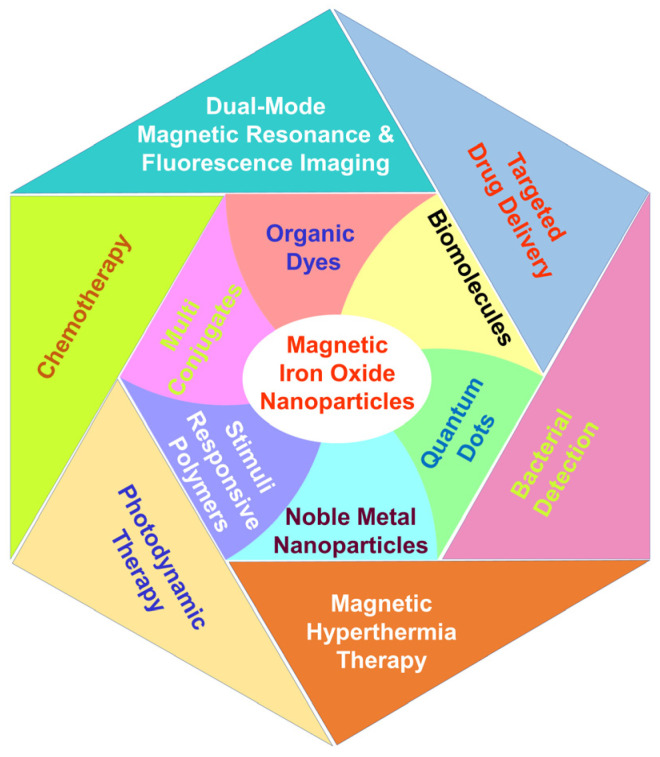
Multifunctional iron oxide NPs integrated with different classes of organic and inorganic materials for biomedical applications.

Nanoparticles (NPs) have attracted substantial scientific attention because they offer novel structural, optical, and electronic properties that are distinct from those of individual molecules or bulk materials. Currently, scientists can design and prepare exotic NPs with controllable sizes, morphologies, and compositions for various applications [1,2,3,4,5]. Among the various types of NPs, magnetic NPs are a promising nanoscale tool in the current biomedical field [5]. For example, the capacity of NPs to generate magnetic fields and influence their local environment has led to their use as contrast agents in magnetic resonance imaging (MRI) techniques [6,7]. Furthermore, their capacity to be manipulated via an external magnetic field makes them attractive candidates for use as drug-delivery vehicles and in cell separation/purification and cell tracking [8,9]. Additionally, their capacity to produce heat when subjected to an oscillating magnetic field makes them suitable as antitumor therapeutic agents [7,9]. Due to their good magnetic properties, excellent biocompatibility, and low cast, magnetic iron oxide nanoparticles (IONPs) are the most commonly used magnetic nanomaterials and have been extensively explored in a wide range of fields, including biomedical, sensing, environmental science, energy storage, and electronic devices [5,6,8,10]. Although magnetic IONPs can be used for a variety of applications in biomedicine, most practical applications require IONP platforms that perform several tasks in parallel. This parallel activity can be achieved by appropriate engineering and integration of magnetic IONPs with suitable conjugates, rendering them practical for use in a broad spectrum of biomedical fields. Moreover, attaching appropriate organic molecules (e.g., dyes, polymers, proteins, and/or antibodies, or other nanomaterials, such as quantum dots and noble metal NPs) to magnetic IONPs allows new biological applications, including protein purification and biosensing [1,8]. In this review, we focus on the progress in current composite NPs based on the integration of magnetic IONPs with different classes of organic and inorganic materials. The integrated materials include organic dyes, biomolecules (e.g., lipids, DNAs, and folic acid), quantum dots, noble metal NPs (including Au and Ag NPs), and stimuli-responsive polymers. We will give a brief overview and highlight the recent technological advances achieved in these integrated multifunctional platforms and their potential use in biomedical applications, as shown in Figure 1.

## 2. Integration of Organic Dyes and Magnetic Iron Oxide Nanoparticles

Magnetic NPs are commonly used as contrast agents in magnetic resonance imaging (MRI) [5,6,9], which provides physiological or biochemical information on what is happening inside the body with high spatial resolution and unlimited imaging depth. Organic dyes, such as fluoresceins, rhodamines, and cyanine [11,12], are a class of contrast agents that are commonly exploited in fluorescence imaging. Fluorescence imaging (FI) techniques provide excellent resolution, high sensitivity, and fast scan times; however, they can suffer from a shallow tissue penetration depth [13,14]. Given these considerations, combined MRI and FI imaging systems have been developed, which offer the combined benefits of these two techniques [15,16,17,18,19,20,21,22,23,24,25,26,27,28,29,30,31]. Many researchers have extensively studied the integration of magnetic NPs and organic dyes into single platforms and have demonstrated their use as bimodal imaging agents for both in vitro and in vivo imaging [15,18,19,20,21,24,25,26,27,28,29,30,31] and in multifunctional platforms that perform several tasks in parallel (e.g., dual-mode imaging and photodynamic therapy or drug delivery) [16,17,22,23]. Several representative studies on the integration of organic dyes and magnetic IONPs for biomedical applications covered in this section are compiled in Table 1, and SPIONs corresponds to superparamagnetic iron oxide nanoparticles.

**Figure 2:**
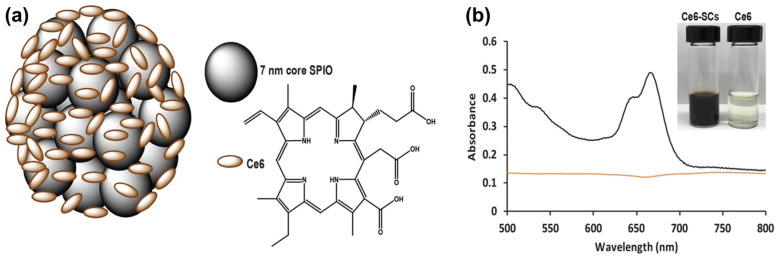
(**a**) Ce6-coated SPION nanoclusters (Ce6-SCs) as a theranostic dual-mode imaging agent and photodynamic therapy agent. (**b**) Absorbance spectra of free Ce6 (brown) and Ce6-CSs (black) in aqueous solutions. Adapted with permission from reference [16].

**Figure 3:**
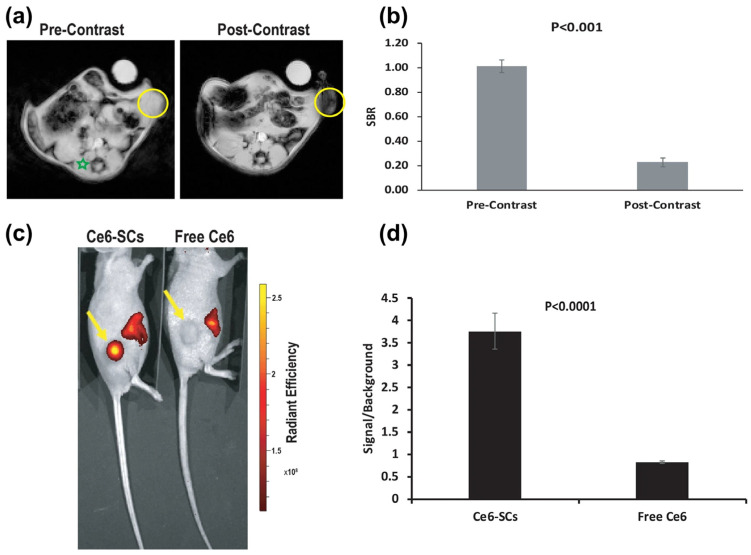
(**a**) In vivo magnetic resonance images (MRI) obtained pre- and 24 h post-intravenous injection of Ce6-SCs into mice bearing 4T1 flank tumors, denoted by yellow circles. (**b**) Quantification of the preinjection vs. postinjection signal-to-background ratio (SBR) measured using the candidate tumor and the paraspinous musculature (green star) background. (**c**) Fluorescence images (FIs) of mice acquired 24 h following injection of 2.5 mg/kg Ce6 based on the Ce6 weight of Ce6-SCs (left) and free Ce6 (right). (**d**) Signal-to-background ratio (SBR) of mice injected with Ce-SCs and free Ce6 at 640 and 720 nm excitation and emission, respectively. Reproduced with permission from reference [16].

Recently, Tsourkas and coworkers combined superparamagnetic SPIONs and chlorin e6 (Ce6), a second-generation and clinically used photosensitizer, to develop a theranostic agent for dual-mode imaging and photodynamic therapy [16]. Photodynamic therapy (PDT) is a relatively new modality based on a minimally invasive procedure for spatiotemporally selective treatments for cancer and other malignant diseases [32,33]. PDT uses light, chemical photosensitizers (PSs), and molecular oxygen or other adjacent substrates to generate cytotoxic reactive oxygen species (ROS) that eradicate target tumor cells. As shown in Figure 2a, Ce6-coated SPION nanoclusters (Ce6-SCs) were synthesized by mixing Ce6 and SPIONs in an oil-in-water emulsion [16]. Due to the presence of four nitrogen atoms and three carboxylic acid groups in each Ce6 molecule, Ce6-SCs are highly soluble and stable in water and can be excited with an ~670 nm laser (Figure 2b). Moreover, Ce6-SCs also showed good biocompatibility, high solubility, and stability under physiological conditions. Importantly, tumors can be detected and visualized by both MRI and FI due to enhanced permeability and retention of Ce6-SCs in tumors compared to free Ce6, as shown in Figure 3. Figure 3 also shows that Ce6-SCs significantly slowed tumor growth in mice due to high singlet oxygen generation during PDT treatments [16].

**Figure 4:**
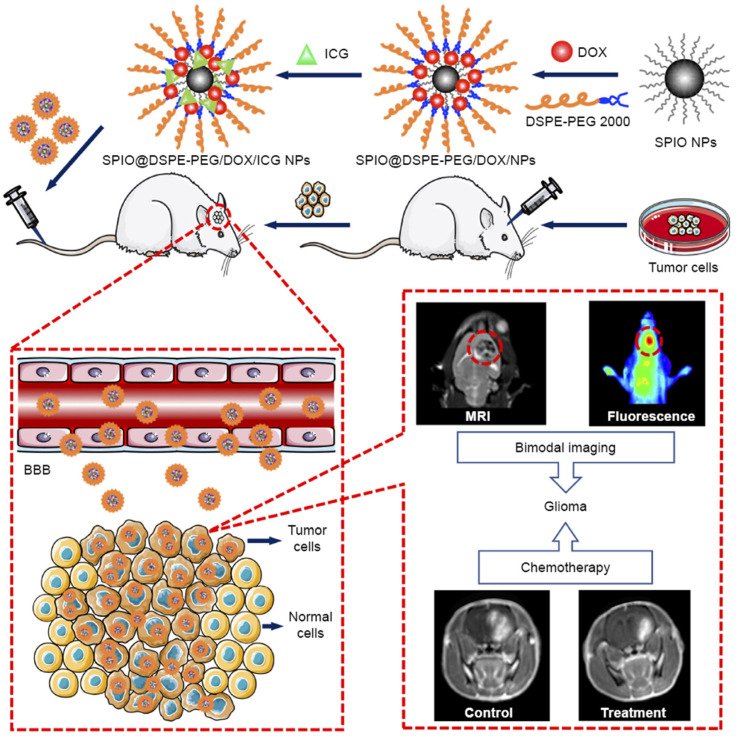
Illustration of the preparation and utilization of SPIO@DSPE-PEG/DOX/ICG NPs in MR/NIR fluorescence dual-modal imaging and chemotherapy of glioma through intravenous injection. Reproduced with permission from reference [17]. Copyright 2019 Dove Medical Press Limited.

In 2019, a multifunctional nanotheranostic agent, SPIO@DSPE-PEG/DOX/ICG nanoparticles, was developed by Dai and coworkers, who simultaneously loaded the traditional chemotherapeutic doxorubicin (DOX) and the organic dye indocyanine green (ICG) into 1,2-distearoyl-sn-glycero-3-phosphoethanolamine-*N*-[methoxy(polyethylene glycol)-2000]-coated SPIONs [17]. The hydrophobic SPIONs were coated with DSPE-PEG 2000 to form a phospholipid shell layer surrounding the SPION core, with the DSPE moieties and hydrophilic outside surfaces derived from the PEG moieties of DSPE-PEG 200. While the lipid shell layer enabled high loading of hydrophobic ICG and DOX, the hydrophilic outside surface ensured high water solubility, excellent biocompatibility, and increased cellular uptake of the NPs [17]. Both in vivo near-infrared (NIR) fluorescence imaging and MRI showed slow and sustained release of DOX from the SPIO@DSPE-PEG/DOX/ICG NPs within tumor cells, resulting in high antitumor efficacy against C6 glioma in rats, without obvious side effects. Figure 4 illustrates the preparation and utilization of SPIO@DSPE-PEG/DOX/ICG NPs in MR/NIR fluorescence dual-modal imaging and chemotherapy of glioma through intravenous injection.

**Scheme 1:**
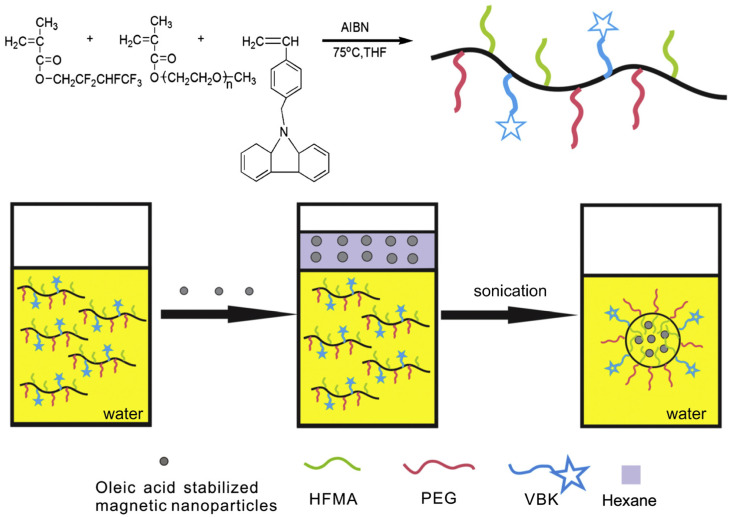
Synthesis of the magnetic fluorescent polymeric micelles Fe_3_O_4_@poly(HFMA-co-VBK)-g-PEG for magnetic resonance and optical imaging. Reproduced with permission from reference [19]. Copyright 2014 Elsevier.

Iron oxides and organic dyes can also be integrated into micelle-based nanoparticles for biomedical applications. Specifically, Yan et al. reported the fabrication of Fe_3_O_4_-encapsulated polymeric micelles via self-assembly of fluorine-containing amphiphilic poly(2,2,3,4,4,4-hexafluorobutyl methacrylate-co-9-(4-vinylbenzyl)-9H-carbazole)-g-poly(ethylene glycol) copolymers and oleic acid-modified Fe_3_O_4_ NPs for dual-modality magnetic resonance and optical imaging [19]. Due to the presence of the magnetic core and fluorescent carbazole dyes in the polymeric shell, the Fe_3_O_4_@poly(HFMA-co-VBK)-g-PEG micelles performed well as a dual-imaging probe with enhanced contrast and blue fluorescence in the liver and spleen during in vivo MRI and optical imaging, respectively. The preparation of the Fe_3_O_4_@poly(HFMA-co-VBK)-g-PEG micelles is illustrated in Scheme 1.

**Scheme 2:**
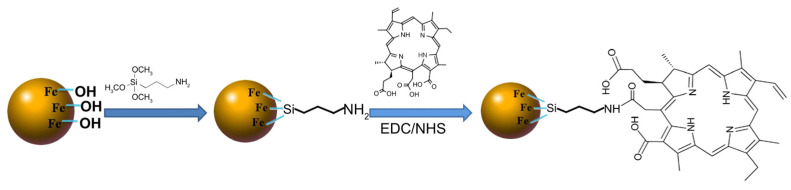
Illustration of the preparation of Ce6-MNPs for simultaneous targeted photodynamic therapy (PDT) and in vivo dual-mode NIR fluorescence imaging and MRI. Reproduced with permission from reference [22]. Copyright 2011 Elsevier.

**Figure 5:**
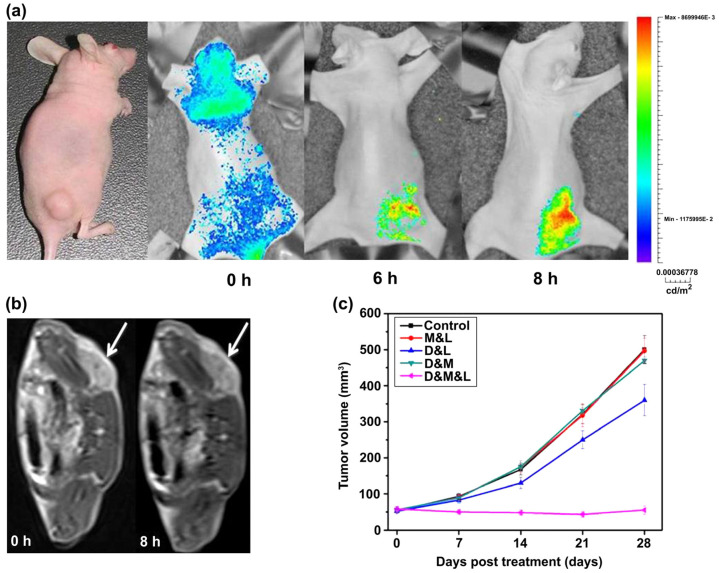
(**a**) In vivo NIR fluorescence images, (**b**) in vivo MRI images, and (**c**) photodynamic efficacy after intravenous injection of Ce6-MNPs in nude mice. Abbreviations: M&L, magnetic targeting and irradiation; D&L, Ce6-MNPs administered and irradiated; D&M, Ce6-MNPs administered and magnetic targeting; and D&M&L, Ce6-MNPs administered, magnetic targeting, and irradiated. Reproduced with permission from reference [22]. Copyright 2011 Elsevier.

Photosensitizer-conjugated magnetic NPs with a diameter of ~20 nm were prepared by Cui and coworkers for simultaneous in vivo gastric cancer magnetofluorescence imaging and targeted therapy [22]. Fe_3_O_4_ NPs were coated with 3-aminopropyltrimethoxysilane (APTS), followed by conjugation of chlorin e6 (Ce6) via a carbodiimide/*N*-hydroxysuccinimide (EDC/NHS) reaction to form Ce6-MNPs (Scheme 2). The as-synthesized Ce6-MNPs were highly soluble in water, noncytotoxic, and biocompatible. Importantly, while the magnetic core allowed MRI, the Ce6 dye offered near-infrared (NIR) FI and photodynamic therapy (PDT) functions. Consequently, Ce6-MNPs were effective for simultaneous in vivo targeted photodynamic therapy, as well as dual-mode MRI and NIR FI of nude mice bearing gastric cancer [22], as shown in Figure 5.

**Scheme 3:**
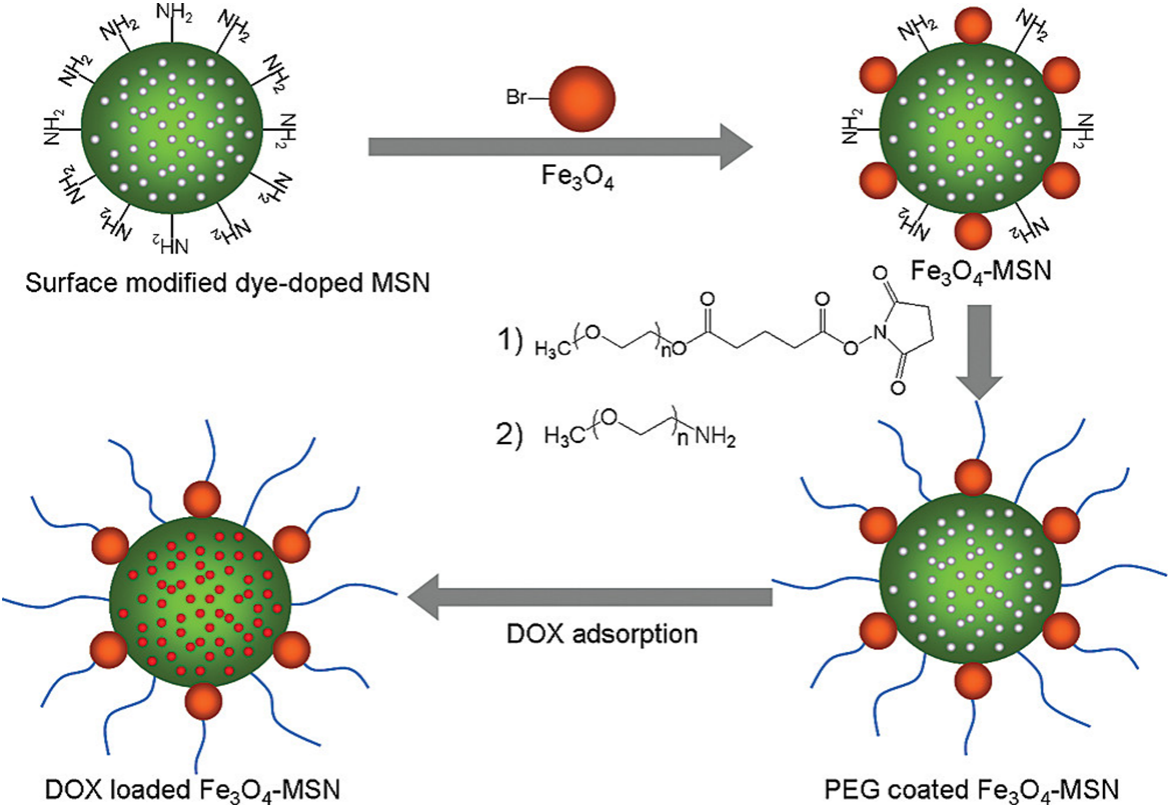
Schematic illustration of the synthesis of DOX-loaded PEG-coated Fe_3_O_4_-MSNs for simultaneous enhanced MRI, FI, and drug delivery. Reproduced with permission from reference [23]. Copyright 2010 American Chemical Society.

In addition to polymer-based structures, silica-based [28] and silica/polymer-based NPs [23,24] have been integrated with organic dyes and magnetic iron oxides for biomedical applications. Hyeon and collaborators described the synthesis of uniform mesoporous dye-doped silica NPs decorated with multiple Fe_3_O_4_ nanocrystals for use as contract agents for simultaneous enhanced MRI, FI, and delivery of the anticancer drug doxorubicin (DOX) [23]. Specifically, the surface of the rhodamine B (RITC)- or fluorescein isothiocyanate (FITC)-doped mesoporous silica NPs was functionalized with APS before reaction with 2-bromo-2-methyl propionic acid-coated Fe_3_O_4_ NPs, followed by surface modification with methoxy poly(ethylene glycol) succinimidyl glutarate (mPEG-SG) and methoxy poly(ethylene glycol) amine (mPEG-AM) to form PEG-coated Fe_3_O_4_-MSNs. DOX was subsequently loaded into the pores of the PEG-coated Fe_3_O_4_-MSNs to generate DOX-loaded PEG-coated Fe_3_O_4_-MSNs. Scheme 3 illustrates the synthesis of the DOX-loaded PEG-coated Fe_3_O_4_-MSNs [23].

**Figure 6:**
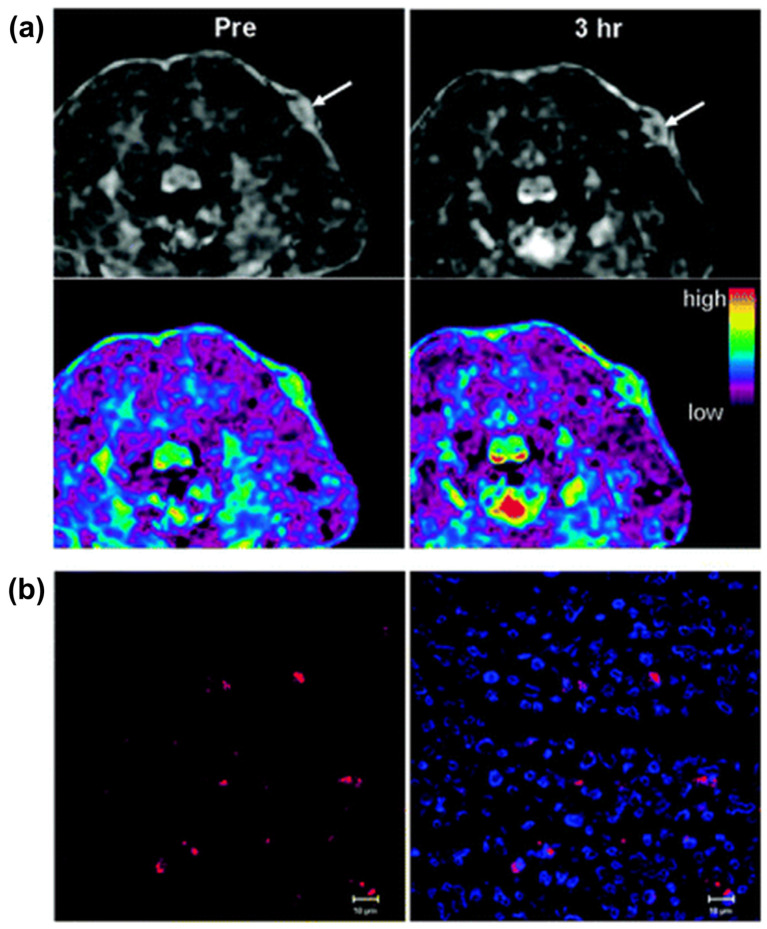
(**a**) In vivo MR images (upper) and color mapped images (lower) of the tumor site (indicated by arrows) before and 3 h after intravenous injection of PEG-coated Fe_3_O_4_-MSNs. (**b**) Confocal laser scanning microscopy images of sectioned tumor tissue harvested 24 h after intravenous injection of RITC-doped PEG-coated Fe_3_O_4_-MSNs (left) and merged with DAPI-stained nuclei (blue, right). Reproduced with permission from reference [23]. Copyright 2010 American Chemical Society.

The synthesized dye-doped PEG-coated Fe_3_O_4_-MSNs exhibited high stability in aqueous solutions and did not show detectable effects on the viability and proliferation of cells. The composite NPs accumulated at the tumor site after intravenous injection. Importantly, the presence of a multitude of magnetic Fe_3_O_4_ NPs on silica surfaces resulted in strongly enhanced MR signals, and the integrated dye molecules in the silica frameworks allowed FI functionality of the dye-doped PEG-coated Fe_3_O_4_-MSNs in vivo [23], as shown in Figure 6. Moreover, DOX was successfully delivered by the platform to the tumor site and induced efficient cancer cell death [23].

The highlighted examples and composite NP systems summarized in Table 1 demonstrate that the integration of iron oxide NPs and organic dyes offers important advantages. Specifically, these platforms can provide better diagnostic information with a more complementary dataset obtained from dual-diagnostic MRI/FI modalities than the corresponding isolated imaging techniques. While the magnetic component can facilitate localization in tumor tissues for MRI in the preoperative stage, the resection of tumors during surgery can be guided by fluorescence imaging. Thus, the combination of magnetic iron oxide NPs and organic dyes can improve the accuracy of diagnosis and reduce damage to healthy tissue, the time needed for testing, the expense, and the uncertainty associated with multiple agents. Moreover, simultaneous multifunctional platforms that perform several tasks in parallel, such as dual-mode imaging and photodynamic therapy or drug delivery, can be achieved by integration of numerous organic dyes and iron-oxide-based MNPs.

## 3. Integration of Biomolecules and Magnetic Iron Oxide Nanoparticles

High biocompatibility, low cytotoxicity, and low risk of immune rejection make biomolecules good candidates for combination with magnetic iron oxide nanoparticles (IONPs) to develop highly targeted platforms for diagnosis, therapy, and theranostics for many notorious diseases. Depending on targeting applications, biomolecules (such as aptamers, antibodies, and even stem cells) can be integrated with magnetic IONPs to achieve desired results. Table 2 summarizes important recent studies on biomolecule-functionalized magnetic iron oxide nanoparticles for biomedical applications covered in this section.

**Scheme 4:**
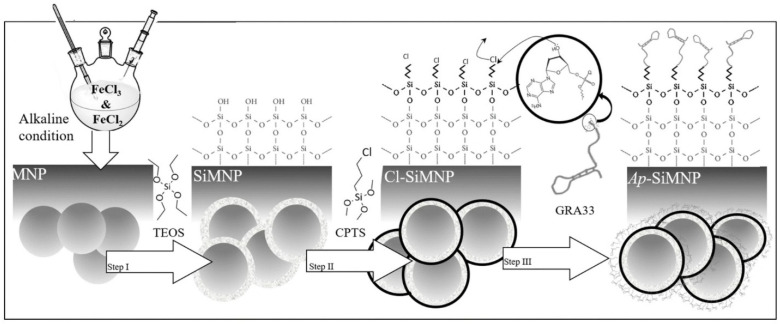
Synthesis and conjugation of SiMNPs with GRA33 DNA aptamers. Reproduced with permission from reference [34].

In recent years, aptamer-enabled technologies have attracted significant attention in the research community as a sensitive, reliable, and convenient method for biomarker detection in various critical human diseases [48]. Combining aptamers and IONPs offers a new set of approaches to develop more effective medical technologies for both diagnostic and therapeutic applications. Taheri and coworkers recently demonstrated that silica-coated IONPs conjugated with DNA aptamers (*Ap*-SiMNPs) can efficiently remove toxic serum albumin prefibrillar amyloid aggregates (AA20) as a potential method to overcome complications related to diabetes [34]. Fe_3_O_4_ magnetic nanoparticles (MNPs) were synthesized using the coprecipitation method, coated with silica, and then conjugated with GRA33 DNA aptamers, as shown in Scheme 4.

**Figure 7:**
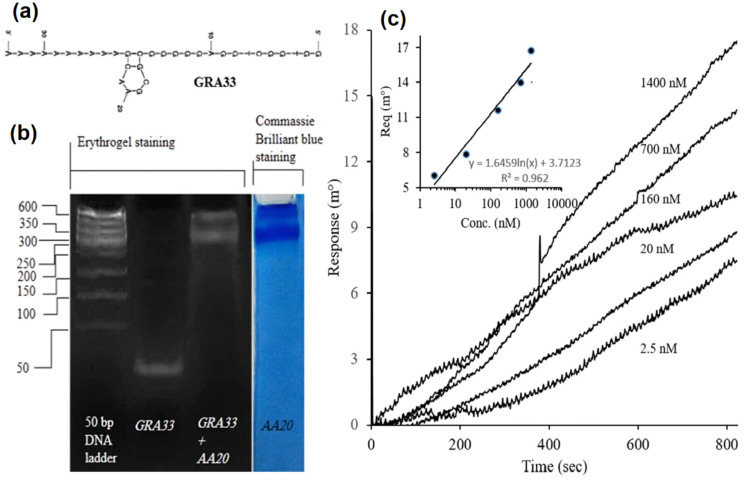
(**a**) DNA sequence of the GRA33 aptamer, (**b**) electrophoretic mobility shift assay results, and (**c**) surface plasmon resonance sensogram results for binding of the GRA33 DNA aptamer with AA20 prefibrillar amyloid aggregates. Adapted with permission from reference [34].

The selective binding of AA20 to the GRA33 DNA aptamer (Figure 7a) was confirmed by an electrophoretic mobility shift assay (EMSA), as shown in Figure 7b. Specifically, GRA33 mobility was strongly retarded after incubation with AA20 compared to GRA33 without AA20. Surface plasmon resonance (SPR) assay results (Figure 7c) also showed the binding of GRA33 to AA20 at various aptamer concentrations. The inset in Figure 7c plots the equilibrium responses used to determine the dissociation constant K_D_ = 3.4 × 10^−9^ M, which represents a high-affinity interaction between GRA33 and AA20. This work provides a promising approach to solve amyloidosis-related complications in diabetic patients and other amyloid disorders.

**Figure 8:**
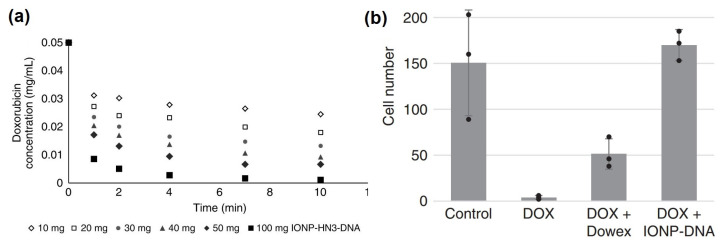
(**a**) DOX concentration in the presence of various amounts of DNA-conjugated IONPs and (**b**) rescue of cultured cells from DOX toxicity by DNA-conjugated IONPs. Adapted with permission from reference [35].

Conventional chemotherapeutic agents used in various medical procedures can cause side effects in patients during and after treatment [49]. Therefore, effective methods to remove excess chemicals from the circulatory system are badly needed to make chemotherapy safer for medical treatment. In 2018, Grubbs and coworkers showed the capture of common chemotherapy agents, including cisplatin, epirubicin (EPI), and doxorubicin (DOX), from biological solutions using genomic DNA-conjugated IONPs [35]. Up to 98% capture of DOX, a commonly used cancer treatment agent, was achieved in human serum within 10 min (Figure 8a). The DNA-conjugated IONPs also captured 20% cisplatin and 68% EPI within 30 min and 25 min, respectively. The authors also demonstrated that their DNA-conjugated nanoparticles could rescue cultured cardiac myoblasts from lethal levels of DOX more efficiently than Dowex, which has previously been shown to reduce DOX levels in vivo (Figure 8b). These results indicate the promising applicability of genomic DNA-conjugated IONPs for drug capture applications to reduce the side effects of chemotherapies.

**Figure 9:**
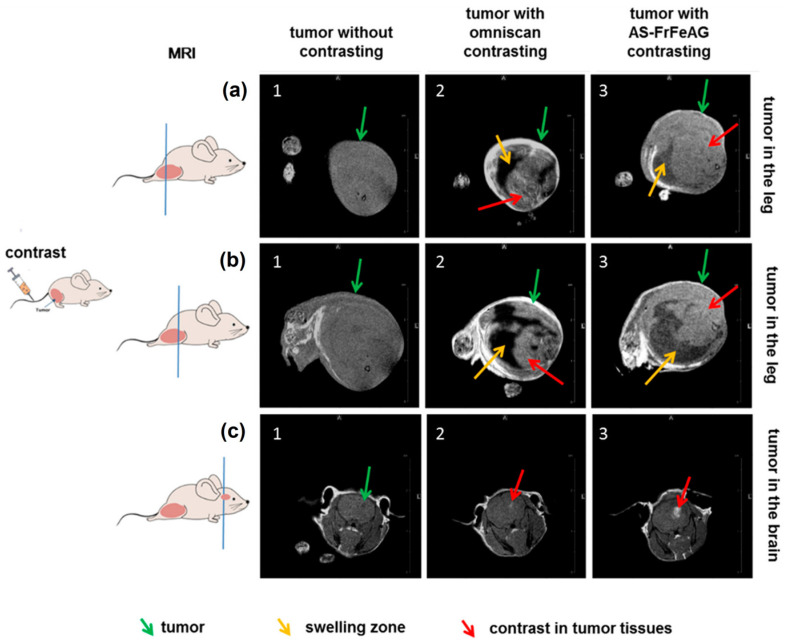
AS-FrFeAG functional nanoparticles used as a contrast agent for magnetic resonance imaging of tumors. A mouse with a tumor transplanted in the right leg (**a**,**b**) or brain (**c**) imaged without a contrast agent (1), with OmniScan as a contrast agent (2), or with AS-FrFeAG as a contrast agent (3). Reproduced from reference [36].

The high toxicity and low efficiency of traditional cancer therapies have been problematic, and nanotechnologies can help with novel solutions. Recently, Kolovskaya et al. reported a magnetodynamic therapy to selectively eliminate tumor cells in vivo [36]. In this work, magnetite nanoparticles functionalized with fibronectin (AS-14), heat shock cognate 71 kDa protein (AS-42) aptamers, and arabinogalactan (AG) polysaccharide were applied for selective cancer cell targeting and cellular internalization. Specifically, the IONPs functionalized with AS aptamers and fragmented AG polysaccharide (AS-FrFeAG) were tested in vivo as a contrast agent for magnetic resonance imaging (MRI) of Ehrlich carcinoma in mice. For tumors in the mouse leg, AS-FrFeAG nanoparticles provided a contrast similar to that of the Omniscan contrast agent (Figure 9a(1–3),b(1–3)). However, the AS-FrFeAG nanoparticles penetrated the blood–brain barrier to reach the brain tumor and yielded a greater contrast than OmniScan (Figure 9c(2,3)), suggesting the potential use of AS-FrFeAG as a contrast agent for brain tumors.

**Figure 10:**
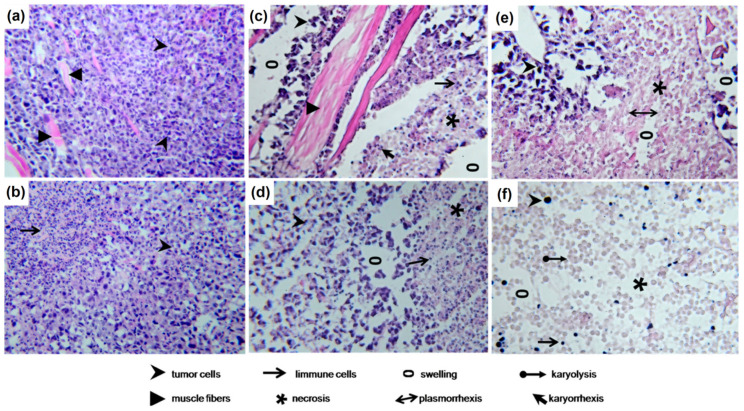
Histological features of the treated tumors: (**a**) nontreated tumor; (**b**) tumor treated with aptamers alone; (**c**,**d**) tumor treated with FrFeAG and LFAMF; (**e**,**f**) tumor treated with AS-FrFeAG NPs under an LFAMF. Magnification ×100. Adapted from reference [36].

AS-FrFeAG nanoparticles were also evaluated for their effect on tumorous cells in the presence of a low-frequency (50 Hz) alternating magnetic field (LFAMF). Compared to untreated tumors (Figure 10a), tumors treated with aptamers alone (Figure 10b), and tumors treated with FrFeAG/LFAMF but without the AS aptamer to target the nanoparticles to the cancer cell (Figure 10c,d), an enhanced reduction in the bulk of the tumor cells was observed for tumors treated with AS-FrFeAG nanoparticles (Figure 10e,f). This work demonstrates the potential of aptamer-functionalized magnetite nanoparticles for noninvasive targeted cancer therapy and contrast enhancement in MRI.

Moreover, with proper aptamer conjugates, magnetic nanoparticles have also been used to enhance the performance of various biomedical applications. Katz and coworkers reported the ability of DNA-modified magnetite nanoparticles to selectively detect and induce downregulation of up to 99% of target mRNA in cultured MCF-7 cancer cells [37]. In a separate work, Fe_3_O_4_@SiO_2_ functionalized with a specific ssDNA aptamer showed highly selective targeting, with a recovery of 87% toward alpha fetoprotein (AFP), a pregnancy biomarker [38]. Zeng and coworkers developed a one-step method to detect thrombin using DNA aptamer-conjugated magnetic nanospheres, with a detection limit of 97 pM [39].

**Figure 11:**
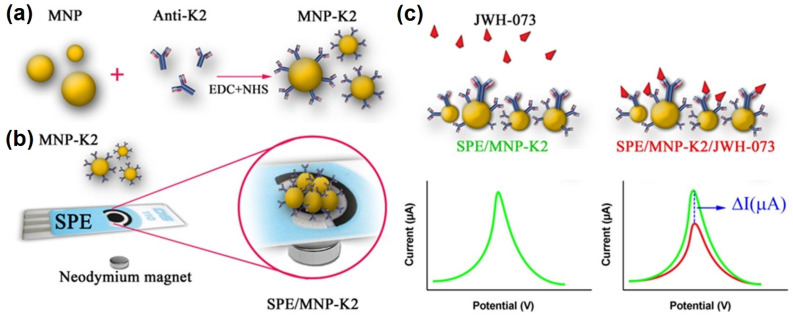
(**a**) Conjugation of MNPs with anti-K2 antibody. (**b**) Capture of MNP-K2 on the screen-printed electrode (SPE) of the sensors via magnetic force. (**c**) Reduction of the electrical current by the presence of JWH-073. Adapted with permission from reference [40]. Copyright 2020 American Chemical Society.

In addition to aptamers, antibodies are another type of biomolecule that is regularly used for many biomedical purposes. Combining various antibodies with magnetic nanoparticles in an appropriate manner can yield a significantly good performance in diagnostics, therapeutics, and theranostics [50]. In a recent report, Timur and coworkers described a biosensor for JWH-073 cannabinoid detection using anti-K2 antibody-immobilized iron oxide magnetic nanoparticles (MNP-K2), as outlined in Figure 11 [40].

**Figure 12:**
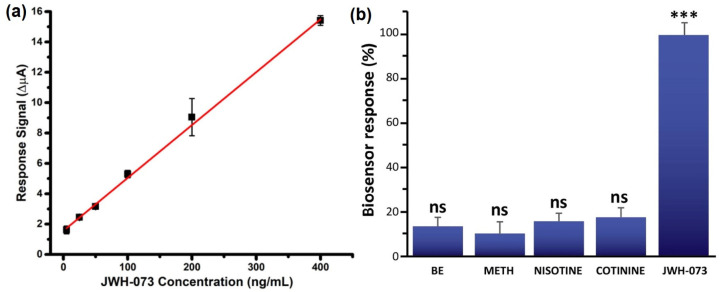
(**a**) Response signal of the MNP-K2 sensor at various JWH-073 concentrations. (**b**) Response of the sensor to certain potential interferents (ns: nonsignificant, *** *p* < 0.001 vs. SPE/MNP-K2). Adapted with permission from reference [40]. Copyright 2020 American Chemical Society.

Testing of the sensing device showed a linear relationship between the response signal and the concentration of JWH-073 used in the test run, as shown in Figure 12a. The high linearity of the signal vs. concentration relationship enables reliable and reproducible detection results, which are important in sensing applications. Moreover, a strong selectivity for JWH-073 by the MNP-K2 sensor was also observed in the presence of possible interferents, such as benzoylecgonine (BE), methamphetamine (METH), nicotine, and cotinine (Figure 12b).

**Figure 13:**
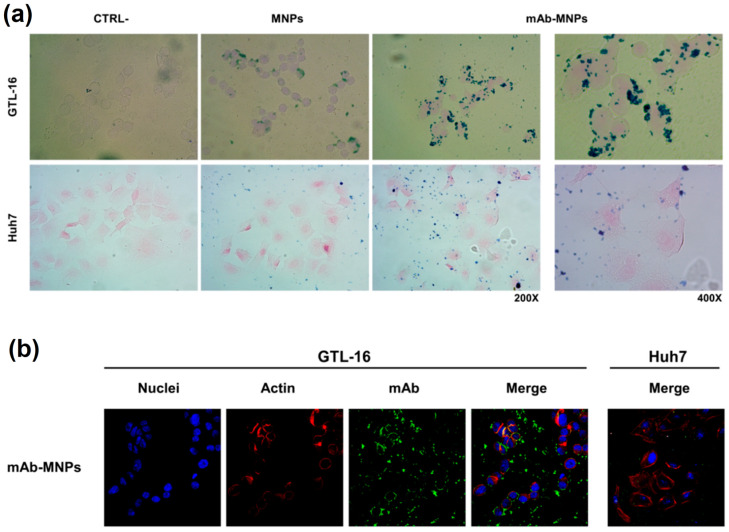
Selective interaction of mAb-functionalized MNPs with GTL-16 cells in comparison with Huh7 cells under (**a**) optical and (**b**) fluorescence confocal scanning microscopy. Adapted from reference [41].

In addition to biomarker detection, antibody-functionalized IONPs have also been used for tumor-targeted drug delivery, as reported in a study by Oltolina et al. [41]. MNPs were functionalized with doxorubicin (DOX, a common chemotherapeutic drug, and with anti-Met/HGFR-positive antibody (mAb), which targets Met/HGFR-positive GTL-16 xenotumor cells. The mAb-functionalized MNPs displayed selective adsorption toward GTL-16 cells compared to Met/HGFR-negative Huh7 cells, as shown in both optical (Figure 13a) and fluorescence (Figure 13b) micrographs.

**Figure 14:**
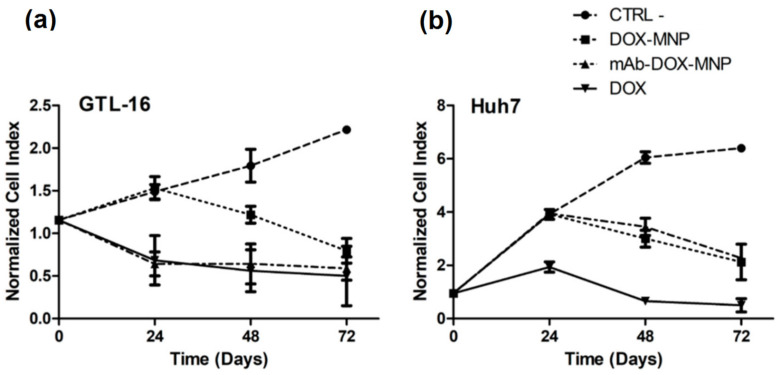
Cytotoxicity of mAb-DOX-functionalized nanoparticles against (**a**) Met/HGFR-positive GTL-16 and (**b**) Met/HGFR-negative Huh7 cells. Adapted from reference [41].

Due to the GTL-16-specific binding of the mAb, the presence of functionalized MNPs (mAb-DOX-MNPs) significantly reduced the viability of Met/HGFR-positive GTL-16 cells (Figure 14a), but no similar decrease in cell viability was observed in Met/HGFR-negative Huh7 cells (Figure 14b). Although the cell viability was roughly similar in the presence of free DOX and mAb-DOX-MNPs, the targeted interaction provided by the mAb can reduce the damaging side effects that are typically observed when using DOX in cancer chemotherapeutic therapies.

Remarkable results in biomedical applications have also been achieved with various antibody-functionalized iron oxide magnetic nanoparticle-based materials. Ramos-Gómez and coworkers demonstrated specific detection of Alzheimer’s disease biomarkers using a nanoconjugate of IONPs and antiferritin antibodies at a nontoxic level [42]. In another work, Wong et al. modified the surface of dextran-coated Fe_3_O_4_ nanoparticles with anti-fetal fibronectin (fFN) antibody to detect the biomarker fFN for sensitive and accurate predictions of the risks of preterm birth [43].

**Figure 15:**
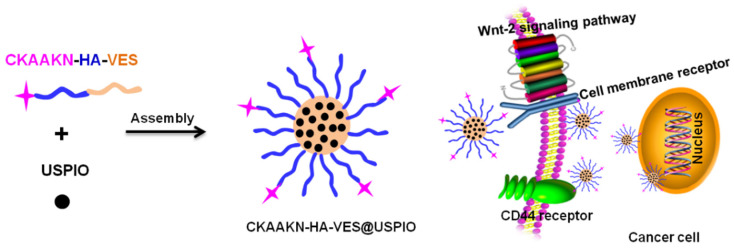
Modification of CKAAKN-HA-VES onto USPIO and illustration of BxPC-3 cancer cell tracking. Adapted from reference [44].

In addition to aptamers and antibodies, various biomolecules have been conjugated with IONPs in research efforts to develop better biomedical technologies or expand the capability of current techniques. In an effort to create a better magnetic resonance imaging (MRI) contrast agent for pancreatic cancer, Zhu et al. grafted a pancreatic cancer-targeting CKAAKN peptide-functionalized amphiphilic hyaluroric acid-vitamin E succinate polymer (HA-VES) onto ultrasmall superparamagnetic iron oxide (USPIO) nanoparticles, as illustrated in Figure 15 [44].

**Figure 16:**
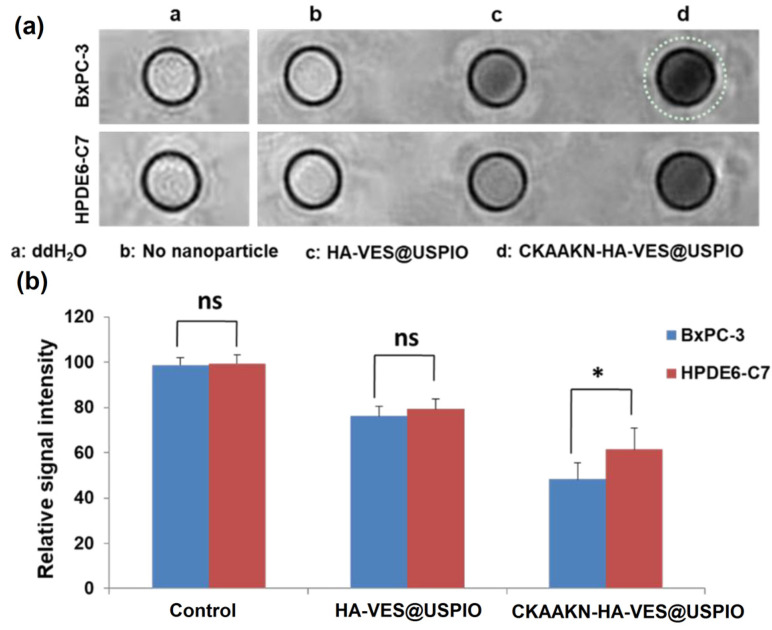
(**a**) In vitro magnetic resonance imaging (MRI) and (**b**) relative MRI signal intensities calculated with ImageJ software of BxPC-3 and HPDE6-C7 cells incubated with magnetic nanoparticles modified with and without CKAAKN (*: ImageJ *p* value < 0.05). Reproduced from reference [44].

The modified CKAAKN-HA-VES@USPIO nanoparticles preferentially internalized into CKAAKN-positive BxPC-3 cells and led to a decrease in MRI signal intensity compared with CKAAKN-negative HPDE6-C7 cells (Figure 16a). As shown in the relative intensity chart in Figure 16b, CKAAKN-HA-VES@USPIO nanoparticles were able to clearly distinguish between BxPC-3 and HPDE6-C7 cells, which led to an enhanced imaging contrast. The work also studied the cytotoxicity of the modified nanoparticles, which resulted in a cell survival rate of over 80% for both BxPC-3 and HPDE6C7 cells after 48 h of incubation with the CKAAKN-HA-VES@USPIO nanoparticles. These results suggest that CKAAKN-HA-VES@USPIO nanoparticles have the potential as an enhanced MRI contrast agent with high specificity and low toxicity for pancreatic cancer diagnosis.

**Figure 17:**
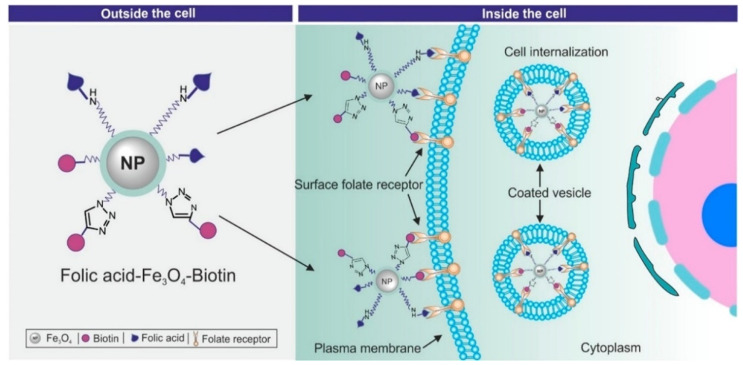
Internalization of the modified magnetic nanoparticles into cancer cells based on multireceptor recognition. Reproduced with permission from reference [45]. Copyright 2020 American Chemical Society.

In another report, Ilyas, Mathur, and coworkers grafted biotin (BT) and folic acid (FA) onto Fe_3_O_4_ magnetic nanoparticles for potential use as biocompatible carriers for anticancer drug delivery or targeting treatment utilizing hypothermal effects from the Fe_3_O_4_ core [45]. The structure combined the targeting abilities of BT and FA with IONPs into a single platform that can molecularly recognize and bind to target cancer cells, as illustrated in Figure 17. Due to molecular identification and internalization by the cell membrane, the modified nanoparticles can enter tumor cells more easily during diagnosis or treatment processes.

**Figure 18:**
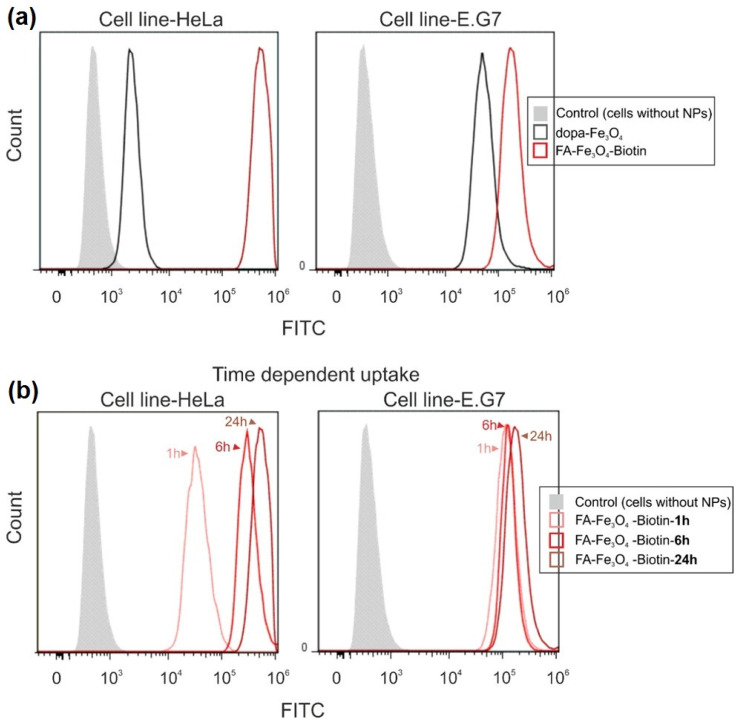
Flow cytometry plots showing the cellular uptake of (**a**) modified and unmodified Fe_3_O_4_ nanoparticles and (**b**) modified nanoparticles over time in HeLa and E.G7 cancer cells. Reproduced with permission from reference [45]. Copyright 2020 American Chemical Society.

Flow cytometry measurements were carried out to evaluate the specific uptake of the modified nanoparticles (FA-Fe_3_O_4_-Biotin) in comparison with the unmodified magnetic nanoparticles (dopa-Fe_3_O_4_) in HeLa and E.G7 cancer cells. Figure 18a reveals that significantly more of the nanoparticles modified with biotin and folic acid than nonmodified nanoparticles were taken up by both cancer cell lines. The functionalized particles also showed higher cell uptake with increasing incubation time (Figure 18b) and low cytotoxicity, with 98% cell viability after 48 h, indicating the high biocompatibility of the particles reported. Separately, iron oxide-lipidoid core–shell nanoparticles were reported by Clauson et al. for MRI-trackable delivery to lymph nodes in mice and can potentially be used as image-guided immunotherapy agents [46]. Kuo et al. reported peptide-functionalized aluminum-oxide-coated IONPs for capture of Staphylococcus aureus, a Gram-positive pathogenic bacterium that can cause food poisoning or infectious disease [47]. Hence, various recent works have demonstrated many designs and combinations of various biomolecules and magnetic iron oxide nanoparticles for advanced biomedical applications.

## 4. Integration of Quantum Dots and Magnetic Iron Oxide Nanoparticles

Quantum dots (QDs), such as CdSe, ZnS, and CdS QDs, and carbon dots (CDs) have been recognized for their unique optical properties. For example, their resistance to photobleaching and their molar extinction coefficient are greater than those of traditional organic dyes; thus, they offer long lifetimes and high brightness [51,52,53,54]. Importantly, their emission spectra can be tuned from the visible to NIR regions by controlling their size and chemical composition [51,52,53,54]. These unique properties have led to great interest in the use of QDs in biomedical applications. To enhance their functionality, QDs are often incorporated with magnetic NPs to produce composite NPs that exhibit both fluorescence and magnetism [55,56,57,58]. These integrated properties allow the development of suitable platforms, which can be examined in vitro before moving to in vivo tests [58,59]. Table 3 lists representative studies on QD-integrated magnetic iron oxide nanoparticles for biomedical applications covered in this section.

**Figure 19:**
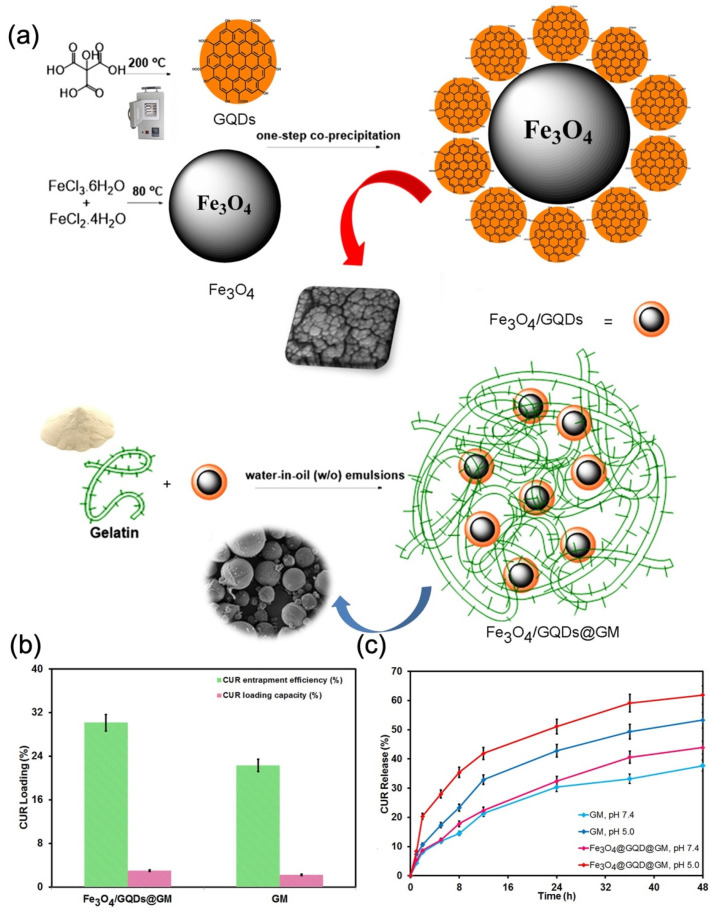
(**a**) Schematic synthesis of Fe_3_O_4_/GQDs@GMs. (**b**) Values of loaded CUR (%) and (**c**) CUR release profiles of GM@CUR and Fe_3_O_4_/GQDs@GM@CUR microspheres at 37 °C under two different pH conditions (5.0 and 7.4). Adapted with permission from reference [64]. Copyright 2020 Elsevier.

Recently, the Namazi group developed gelatin-coated Fe_3_O_4_/graphene QD hybrid microspheres (Fe_3_O_4_/GQDs@GM) for anticancer drug delivery, as shown in Figure 19a [64]. The authors used carbon-based graphene quantum dots (GQDs) due to their excellent biocompatibility, high crystallinity, and fluorescence properties combined with their high density of hydroxyl (−OH) and carboxylic (−COOH) groups on the QD surface. Consequently, the combination of Fe_3_O_4_ and GQDs provides opportunities for loading of abundant types of anticancer drugs in suitably designed microspheres. According to Figure 19b, the synthesized hybrid Fe_3_O_4_/GQDs@GMs revealed a higher loading capability of the well-known anticancer drug curcumin (CUR) than the pure gelatin microspheres (GMs). CUR release studies for both materials are presented in Figure 19c, showing that the release of CUR depended on the pH. The data also showed that the Fe_3_O_4_/GQDs@GMs are better than pure GMs for CUR delivery at both of the studied pH conditions.

Ou et al. reported magnetic Fe_3_O_4_/SiO_2_/graphene–CdTe QDs/chitosan nanocomposites (FGQCs) as a promising multifunctional drug delivery system for biological and medical applications [61]. The CdTe QDs embedded inside the graphene provided the fluorescence capability for traceable imaging to track and diagnose the effectiveness of treatments. While the graphene shells showed good drug-loading capability due to their noncovalent π–π stacking, the SiO_2_ layers prevented fluorescence quenching by blocking direct contact of fluorophores with the magnetic iron oxide NPs.

**Figure 20:**
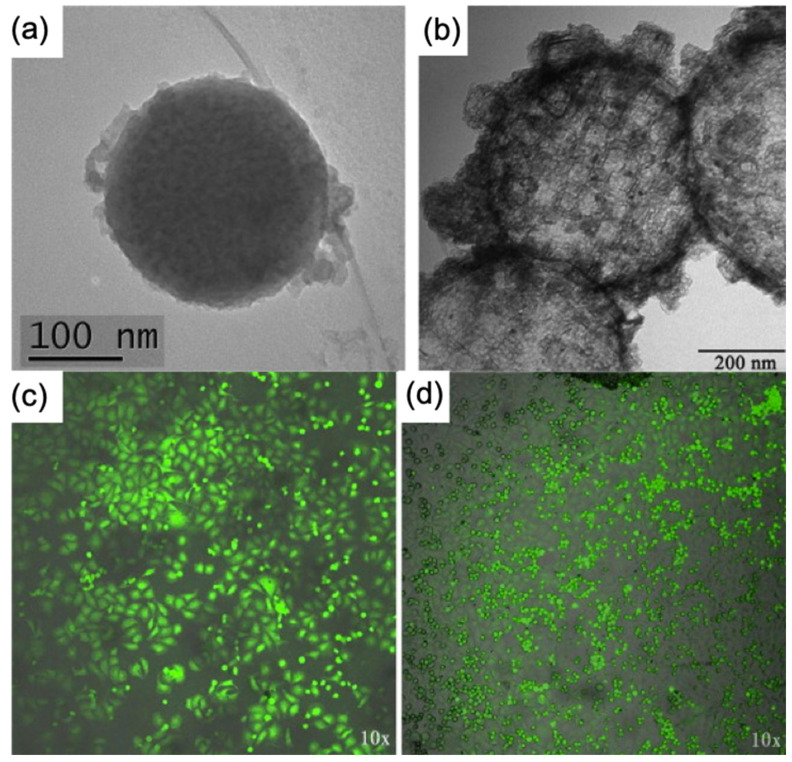
TEM images of (**a**) Fe_3_O_4_@SiO_2_ NPs and (**b**) FGQCs. Laser scanning confocal microscopy in vitro bioimaging of (**c**) FGQC NPs and (**d**) 5-FU-FGQCs in the hepatoma cell line SMMC-7721. Adapted with permission from reference [61]. Copyright 2014 Elsevier.

The morphologies of both Fe_3_O_4_@SiO_2_ and FGQCs are presented in Figure 20a,b. The size of FGQC increased to ~460 nm after coating the ~220 nm Fe_3_O_4_@SiO_2_ NPs with graphene–CdTe QD shells. Fluorouracil (5-FU) was used as the drug sample in this study and showed 70% loading content and 50% entrapment efficiency. By comparing the results presented in Figure 20c,d, it can be observed that significant growth inhibition of the hepatoma cell line SMMC-7721 occurred. The authors believe that the growth inhibition of the hepatoma cell line SMMC-7721 was induced by 5-FU-FGQCs at ~1 μg/mL (the IC_50_ concentration was 50% compared to only 10% of the same amount of free 5-FU). Hence, FGQCs, with magnetic and fluorescence characteristics, have potential for drug delivery applications.

Despite these promising results, we note that the main concern regarding the use of many magnetic-QD platforms in biomedical applications is their inherent toxicity, which arises from the heavy metals in QDs (e.g., Cd, Se, Pb, As, and/or In) [65,66]. Consequently, applications involving these magnetic-QD platforms are currently restricted to in vitro and animal studies.

## 5. Integration of Noble Metal Nanoparticles and Magnetic Iron Oxide Nanoparticles

Noble metal nanoparticles exhibit remarkable optical properties that arise from surface plasmon resonances, which can be controlled from the ultraviolet to the near-infrared (NIR) regions of the electronic spectrum by optimizing the size, composition, shape, and topology of noble metal NPs [67,68,69,70]. As NIR light can penetrate deeply into human tissue [71], the absorption of light in the NIR regions by noble metal NPs makes them great candidates for use as contrast imaging agents to visualize organ tissue [72] and as therapeutic agents for tumor ablation and/or drug delivery [73]. The combination of noble metal NPs and magnetic NPs into one platform offers multiple diagnostic and therapeutic modalities simultaneously, which can improve the accuracy and clarity of diagnostic images while reducing time and expense [74]. Table 4 summarizes representative studies on the integration of noble metal NPs and magnetic NPs for biomedical applications covered in this section.

Due to their optical properties, stability, and biocompatibility, gold nanoparticles (Au NPs) have been used in various biological applications. Iron oxide and gold-based nanostructures for medical applications have been reviewed in the literature [81]. In general, magnetoplasmonic nanoassembly hybrid systems combine the advantages of the magnetic and plasmonic properties of Fe_3_O_4_ and Au NPs. For example, Li et al. reported the use of Fe_3_O_4_@Au-mPEG-PEI core–shell composite nanoparticles for dual-mode MR/CT imaging applications [77]. In this system, the Fe_3_O_4_@Au NPs were stabilized with polyethyleneimine (PEI) and poly(ethylene glycol) monomethyl ether (mPEG). A hemolytic assay showed excellent hemocompatibility of Fe_3_O_4_@Au-mPEG-PEI core–shell nanoparticles in the concentration range of 0−400 μg/mL. Additionally, the nanoparticles showed good cytocompatibility at concentrations up to 100 μg/mL. Importantly, the composite NPs exhibited a relatively high r_2_ relaxivity of ~146 mM^−1^S^−1^ and good X-ray attenuation properties. Consequently, the nanoparticles were successfully used as a contrast agent for dual-mode MR/computed tomography (CT) imaging of tumor cells in mouse and rat livers.

Coating of gold on magnetic nanoparticles has been reported to not only enhance biocompatibility but also maintain the magnetic properties of the original NPs. Moreover, the high stability of gold can prevent decomposition of the magnetic nanoparticles [82]. Recently, Multari et al. synthesized hybrid nanoparticles of iron oxide decorated with gold (Fe_3_O_4_-Au) nanoparticles using tannic acid as a reducing agent [75]. The nanoparticles exhibited superparamagnetic behavior and a plasmonic peak at 560 nm, which is suitable for use in biomedical applications. The hybrid Fe_3_O_4_-Au nanoparticles showed good photothermal therapy ability against cancer cells under laser irradiation. Other shapes of gold-based magnetoplasmonic nanoparticles have been developed for use in biological applications. For example, Zhou et al. reported an in vivo study of spiky Fe_3_O_4_@AuNPs [76]. Specifically, spiky Fe_3_O_4_@AuNPs with different branch lengths exhibited different SPR peak positions. While short-branched NPs with 51.4 nm cone-shaped AuNPs on spherical Fe_3_O_4_ surfaces showed an SPR peak at 575 nm, long-branched NPs exhibited two extinction peaks of transverse and longitudinal modes at 540 and 745 nm, respectively [76]. Moreover, both short- and long-branched spiky Fe_3_O_4_@AuNPs exhibited low toxicity and good biocompatibility, which are important for applications of nanoparticles as theragnostic agents.

**Figure 21:**
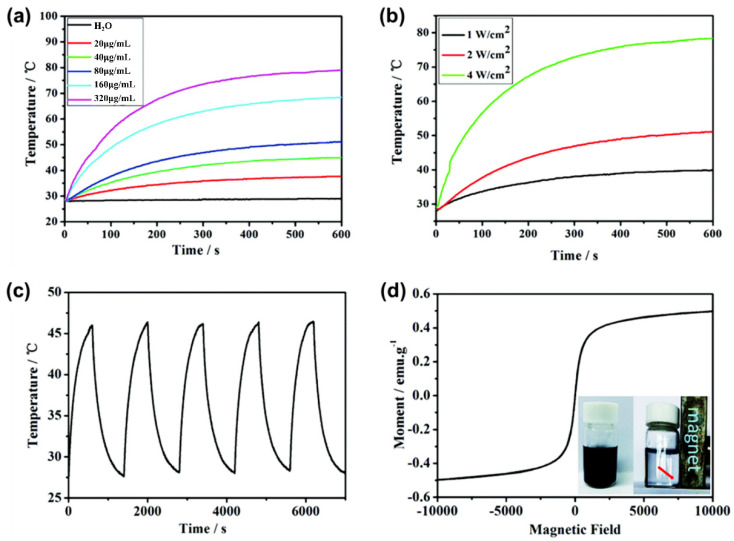
(**a**) Temperature profiles of ultrapure water and PMGNS solutions at different concentrations as a function of 808 nm laser irradiation for 10 min at a power density of 2 W cm^−2^. (**b**) Temperature profiles of PMGNS solutions at different powers as a function of 808 nm laser irradiation for 10 min at a concentration of 80 μg mL^−1^. (**c**) Temperature monitoring of a PMGNS aqueous suspension at a concentration of 80 μg mL^−1^ during successive ON/OFF laser cycles at a power density of 2 W cm^−2^. (**d**) Magnetic hysteresis curves of PMGNSs attracted by a magnet. Reproduced with permission from reference [78].

Hou and coworkers synthesized theranostic agents based on Fe3O4@SiO2@GNS–PEG (PMGNS) nanoparticles [78]. The PMGNS nanoparticles showed imaging contrast abilities in both magnetic resonance (MR) and computed tomography (CT) at different NP concentrations. Specifically, the photothermal properties of the aforementioned nanoparticles were evaluated by testing the temperature increase profiles of PMGNS solutions at different concentrations (Figure 21a) and different power densities (Figure 21b) under NIR laser irradiation. The results from both sets of photothermal heating curves showed the highest temperature of 79.1 °C at a concentration of 320 μg/mL. Moreover, the temperature profiles after five cycles of heating were obtained to test the thermal stability of the PMGNS nanoparticles. As shown in Figure 21c, the temperature of the PMGNS aqueous solution still increased after five heating cycles. The magnetization curves obtained using a vibrating sample magnetometer (VSM) showed a saturation magnetization (Ms) value of 0.52 emu g^−1^ for these PMGNSs, as illustrated in Figure 21d.

**Figure 22:**
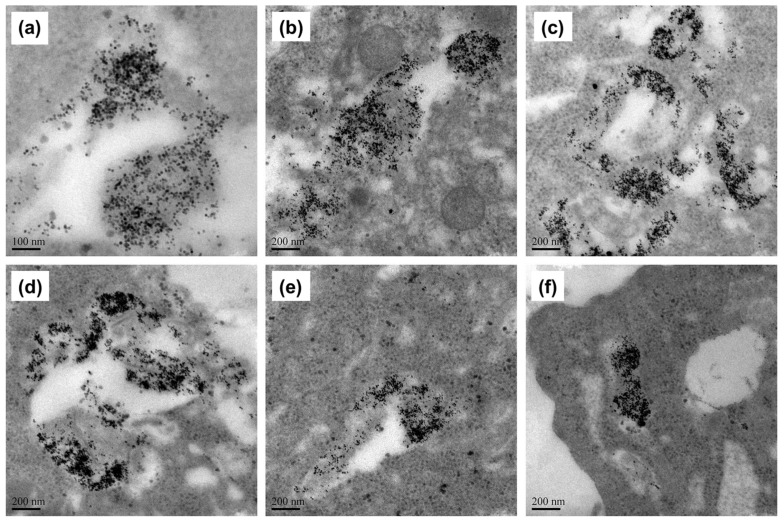
TEM images of SMMC-7721 cells treated with (**a**,**d**) Fe_3_O_4_-PAA, (**b**,**e**) Fe_3_O_4_-Ag-PAA, (**c**,**f**) or Fe_3_O_4_@Ag-PAA NPs for 12 h with and without alternating current magnetic field treatment. Reproduced with permission from reference [79]. Copyright 2017 Elsevier.

In addition to gold nanoparticles, silver nanoparticles (Ag NPs) have also been utilized in various biomedical applications, especially as anticancer or antibacterial agents [83]. Various magnetite-silver hybrid nanoparticles, including core–shell, multishell core–shell, and heteromeric structures, have been reported [79,80]. For example, magnetite-silver hybrid NPs, both core–shell (Fe_3_O_4_@Ag-PAA) and heteromeric (Fe_3_O_4_-Ag-PAA) nanoparticles, were developed for tumor magnetic hyperthermia treatment [79]. Both the Fe_3_O_4_@Ag-PAA and Fe_3_O_4_-Ag-PAA nanoparticles exhibited higher hyperthermia efficiency than bare magnetic nanoparticles. As shown in Figure 22, TEM images of SMMC-7721 cells treated with Fe_3_O_4_-PAA NPs (Figure 22a,d) showed significantly less magnetic hyperthermia efficiency than SMMC-7721 cells treated with Fe_3_O_4_@Ag-PAA (Figure 22b,e) and Fe_3_O_4_-Ag-PAA hybrid NPs (Figure 22c,f) in the presence of an alternating-current magnetic field.

In a separate study, Marmoudi and Serpooshan reported superparamagnetic iron oxide nanoparticle (SPION) core-ultrathin silver shell structures with polymeric ligand gaps for use as multimodal antibacterial agents [80]. Interestingly, SPION-silver core–shell, SPION-gold core–shell, and SPION-gold-silver core-intermediate shell–shell nanoparticles were synthesized, and the properties of the silver and gold shells were compared for therapeutic applications. The silver-coated SPIONs and gold-silver-coated SPIONs showed high therapeutic indices against *Staphylococcus epidermidis* and *Staphylococcus aureus* infections, as determined by live/dead assays. Moreover, the silver-coated SPIONs exhibited less toxicity in a human liver carcinoma cell line (HepG2), confirming the biocompatibility of these functionalized silver nanoparticles.

The examples shown here demonstrate the fabrication and utility of various types of magnetic iron oxide NP nanocomposites integrated with different noble metal NPs. The functionality of the nanocomposites can be tuned by varying the size, composition, shape, and topology of the NPs. Moreover, the examples presented here illustrate that rational engineering and design can transform nanomaterials into practical tools for future biomedical applications.

## 6. Integration of Stimuli-Responsive Polymers and Magnetic Iron Oxide Nanoparticles

Integration of magnetic NPs with polymers can expand the scope of their application in a variety of ways. Polymers have been widely used to improve the stability, aqueous dispersion, biocompatibility, and bioavailability of magnetic NPs for in vivo applications [84]. A common example of this is embedding magnetic NPs inside a shell composed of the US FDA-approved polymer poly(lactic-co-glycolic acid) (PLGA) and its derivatives [84,85,86]. However, the potential of magnetic nanoparticles is truly realized when integrated with stimuli-responsive polymers. Stimuli-responsive polymers can exhibit dramatic property changes in response to one or more external stimuli, such as temperature, pH, light, chemical, electrical, and mechanical stress [86]. Table 5 lists representative studies on stimuli-responsive polymer-integrated magnetic nanoparticles for biomedical applications covered in this section.

### 6.1. Integration of Thermo-Responsive Polymers

**Figure 23:**
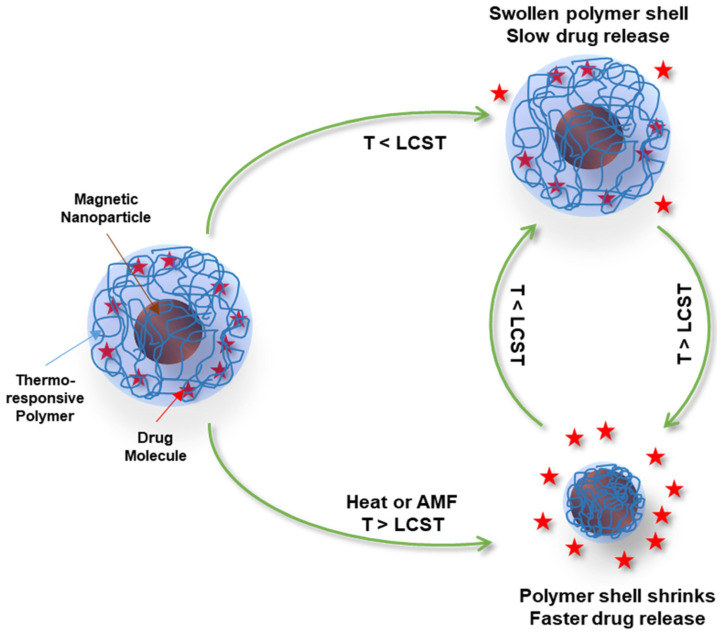
Illustration of the reversible swelling–collapse behavior and drug release from thermoresponsive polymer-magnetic NP composites upon heating or application of an alternating magnetic field (AMF). Adapted with permission from reference [89]. Copyright 2010 Elsevier.

By coupling the unique properties of magnetic NPs and thermally responsive polymers, the emergence of a new paradigm in controlled drug delivery and novel nanoscale therapeutic agents is now a reality. In general, these developed platforms are prepared by the encapsulation/deposition of magnetic NPs within stimuli-responsive polymer networks [98,99,100,101,102,103,104]. The polymeric layer serves as a “smart” container that can encapsulate/load and subsequently release drugs/substances from the polymer network upon external adjustment of the local temperature above the lower critical solution temperature (LCST) of the stimuli-responsive polymer [98,99,100,101,102,103,104]. The magnetic component acts as an energy converter to transfer received magnetic energy to heat via hysteresis losses and Néel and Brownian relaxation effects [105,106]. Hence, an oscillating magnetic field can increase the temperature of polymer networks near magnetic NPs, thereby enabling remotely modulated drug delivery [100,102,103,104]. The process of releasing a drug from these polymer-MNP composites is illustrated in Figure 23.

Poly(N-isopropylacrylamide) (PNIPAm) is one of the most commonly used thermally responsive polymers, with dimensions that can be altered upon stimulation by temperature [107]. Above a certain temperature in aqueous solutions, the hydrogen bonds in such hydrogel polymers are broken, and the polymer undergoes a reversible phase transition from a swollen hydrated state to a collapsed dehydrated state. The temperature at which the phase transition occurs is defined as the lower critical solution temperature (LCST) [108,109,110,111]. The LCST of PNIPAm is approximately 32 °C [112,113]. Localized heating of PNIPAm integrated with plasmonic metal nanostructures has been widely exploited for remote-controlled drug delivery triggered by NIR light [85]. NIR light can pass through human tissues and couple with NIR-responsive nanoparticles, which in turn generate heat in the integrated PNIPAm materials [85]. NIR light-triggered delivery of anticancer drugs has also been achieved using Fe_3_O_4_@PNIPAm yolk-shell NPs [90].

Magnetic nanoparticles offer another important mechanism for remote heating of PINPAM nanoparticles. Heat is generated in magnetic nanoparticles when they are subjected to an alternating magnetic field (AMF), which can lead to deswelling of integrated polymers and the release of encapsulated drugs. Ramanujan and coworkers were among the first groups to integrate PNIPAm with IONPs for targeted release of the anticancer drug doxorubicin (DOX) [88,89]. Preliminary studies showed that DOX can be loaded within a magnetic PNIPAm nanoparticle and subsequently released by either adjusting the local temperature above the LCST or using a rapidly alternating magnetic field [88]. During the course of exposure to a magnetic field (47 min), approximately 14% of the drug was released, and the temperature of the colloidal solution was rapidly increased from room temperature to 41–48 °C. The ability to generate heat in the range of 41–48 °C when subjected to an external magnetic field allows this platform to potentially be used as a therapeutic agent that can simultaneously release drugs and generate heat for cell ablation (e.g., tumor eradication). In a related study, drug release from a PNIPAm-based core–shell magnetic NP was confirmed to be much higher above the LCST, with 78% release of the loaded drug within 29 h [89]. In another example, Jaber et al. prepared IONPs embedded in mesoporous SiO_2_ shells (IONPs@mSiO_2_) and then loaded PNIPAm containing the anticancer drug H_3_PMo_12_O_40_ in the pores of the mesoporous SiO_2_ shells for controlled drug release under an alternating current magnetic field [114]. However, in most of these studies, the LCST of PNIPAm itself was approximately 34–35 °C and was unchanged when integrated with IONPs. Given that the physiological temperature of the human body is approximately 37 °C, a higher LCST is desired to prevent undesired drug release in the absence of an AMF.

**Figure 24:**
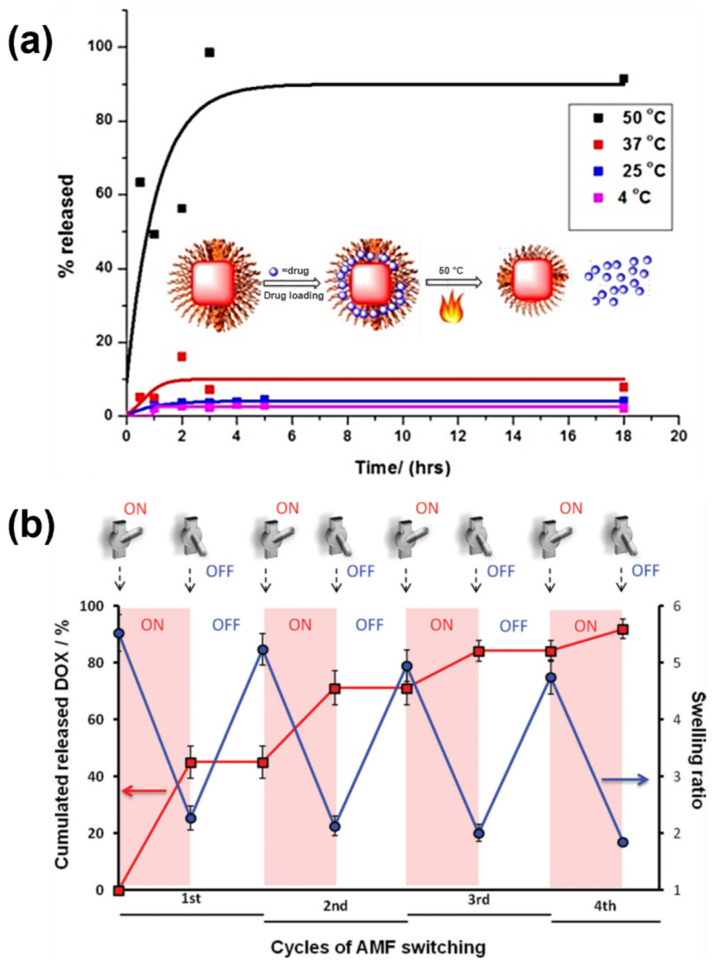
(**a**) Drug release from thermoresponsive PNIPAm-co-PEGA cubic-IONPs in a water bath at various temperatures (50 °C, black box; 37 °C, red box; 25 °C, blue box; 4 °C, purple box). Reproduced with permission from reference [92]. Copyright 2015 American Chemical Society. (**b**) ‘On–off’ switchable and reversible heat profile and swelling ratio of MNPs with crosslinked poly(NIPAm-co-HMAAm) nanofibers and the DOX release profile corresponding to the reversible swell–shrink property in response to temperature changes. DOX release = D released at X/D total × 100 (%); M released at X is the cumulative amount of released DOX at the X cycle of AMF alternation; and M total is the total amount of incorporated DOX in MNP-loaded nanofibers. Swelling ratio = (W_swell_ − W_dry_)/W_dry_, where W_swell_ is the weight of swelled MNP-loaded nanofibers and W_dry_ is the weight of dried MNP-nanofibers. Reproduced with permission from reference [91]. Copyright 2013 John Wiley and Sons.

The LCST of PNIPAm can be increased by increasing the hydrophilic character of the polymer. Chen et al. grew a PNIPAm shell around an IONP@carbon core–shell NP [87]. The presence of multiple functional groups on the carbon shell displayed a ζ-potential of up to -33.0 mV and increased the LCST of the PNIPAm networks to 45 °C. These nanoparticles collapsed from a size of 280 nm to 257 nm under an AMF, effectively releasing the loaded hydrophilic epithelial anticancer drug 5-fluorouracil. Thus, application of an AMF can serve as an “on–off switch” for drug release. Higher LCSTs can also be achieved by cografting other monomers into the PNIPAm chains. Kakwere et al. incorporated the hydrophilic polymer polyethylene-glycolmethyl-ether-acrylate with PNIPAm (PNIPAm-co-PEGMEA) to increase the LCST above 37 °C (see Figure 24a) [92]. In this study, the authors used cubic IONPs, which possess a high specific absorption rate compared to spherical IONPs; as a result, lower doses of IONPs could be applied in vivo to reach therapeutic temperatures. Other commonly used comonomers for increasing the LCST of hydrogel polymers are acryl amide (-*co*-AAm) and acrylic acid (-*co*-AAc); the LCST of PNIPAm-*co*-AAc can be adjusted in a range of ~30–60 °C [115].

Magnetic NPs also have an intrinsic ability to kill cancer cells via magnetic hyperthermia therapy (MHT). AMF-induced MHT is preferred because the tumor temperature can be easily regulated by adjusting the magnetic field strength (H) and frequency (f) [116]. Additionally, AMF can penetrate and heat deep tumors without damaging normal hypodermal tissues [116]. Modern therapeutic materials have been designed to simultaneously exploit both the anticancer drug release and MHT properties of polymer-integrated IONPs, with targeted temperatures above ~45 °C [116]. Aoyagi and coworkers designed DOX- and MNP-embedded hyperthermia nanofibers consisting of a copolymer of NIPAm and N-hydroxymethylacrylamide (HMAAm), named poly(NIPAm-co-HMAAm) [91]. Localized heating to a temperature of 45 °C was achieved, triggering a synergistic activity of DOX release and hyperthermia; as a result, 70% cell death was observed within 5 min. The HMAAm copolymer cross-links provided better stability to the system and prevented MHT side effects from eluted MNPs. Nearly all of the loaded DOX (>90%) was released after four AMF ‘on’ cycles, while only negligible amounts were released during the cooling ‘off’ process (see Figure 24b).

**Figure 25:**
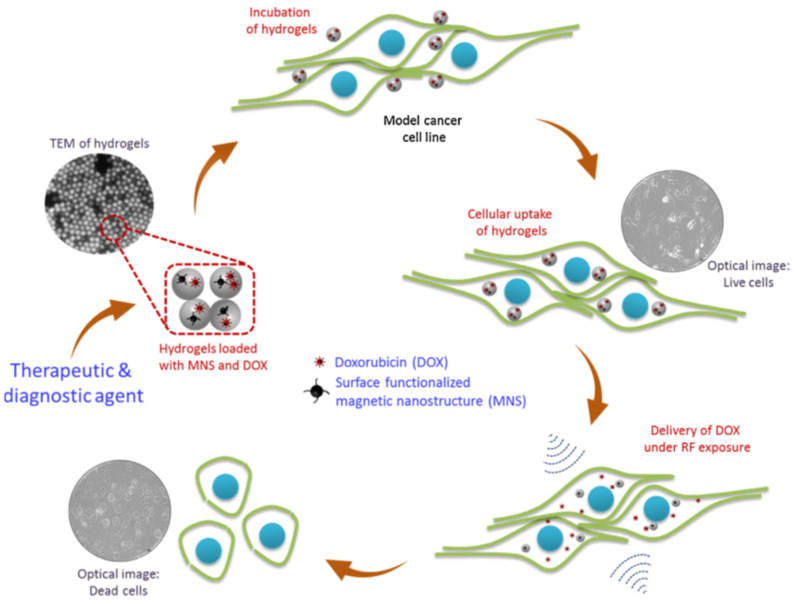
Schematic illustration of the anticancer activity of magnetic nanostructure (MNS) hydrogels loaded with the chemotherapeutic agent doxorubicin (DOX). The MNS was functionalized with nitro-dopamine-PEG to achieve aqueous stability. After cellular uptake, the applied external RF field activates the MNS hydrogels and stimulates DOX release by rupturing the polymeric hydrogel. Due to the presence of the MNS, the hydrogel also acts as an MRI contrast agent. Reproduced with permission from reference [94]. Copyright 2015 Elsevier.

Another important benefit of MNPs is their ability to be viewed via magnetic resonance imaging (MRI), which can enable visual tracking of polymer-integrated MNPs in vivo. Magnetic resonance imaging (MRI)-guided MHT can not only monitor therapeutic outcomes but also measure the tumor temperature without insertion of a thermal probe [93]. Jaiswal et al. embedded PEG-functionalized Fe_3_O_4_ nanostructures in PNIPAm hydrogels, ensuring 95% cancer cell death via the synergistic effects of heating and DOX drug release, as demonstrated in Figure 25 [94]. Importantly, MRI monitoring allowed the authors to study the accumulation of NPs in mouse livers, lungs, and hearts.

**Figure 26:**
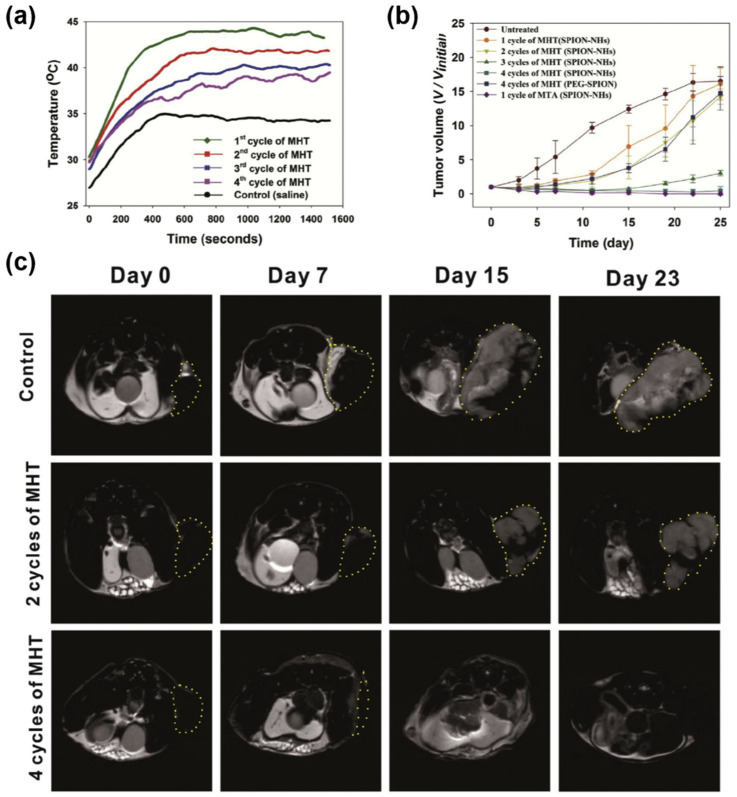
(**a**) Elevated tumor temperature versus time graphs induced by multiple MHT treatments after a single injection of PEGylated SPION aqueous solution. Mice were treated under the same AMF conditions. (**b**) In vivo therapeutic outcomes of multiple MHTs or single MTAs in tumor xenograft mice. Tumor volume (V/V_inital_) was plotted versus time after treatment. (**c**) Long-term MRI monitoring of tumor xenografted mice injected with SPION-NHs. Tumor tissues are marked (yellow dashed line), and the black color within the tumor indicates the presence of SPIONs. Reproduced with permission from reference [93]. Copyright 2016 Elsevier.

Although PNIPAm is the most popular polymer for AMF-triggered drug release, other polymers have also been incorporated with MNPs to perform similar functions. Liu et al. utilized block copolymers of poly(ethylene oxide) and poly(propylene oxide) (PEO-b-PPO-b-PEO) combined with MNPs to trigger an 80% ‘burst’ drug release at 45 °C [95]. The hybrid NPs showed very little leakage at room temperature (25 °C) and physiological temperature (37 °C) [95]. Integrated polymers can also provide additional functions in thermoresponsive magnetic systems, such as long-term retention, cell targeting, and enhanced stability. Zhang et al. used SPION-loaded nanocapsule hydrogels of poly(organophosphazene) (PPZ) to impart long-term retention in tumors (80% after 6 days) for multiple MHT treatments (temp. ~45 °C) while also serving as an MRI contrast agent to guide the therapeutic process [93]. The long-term retention in tumors of PPZ allowed multiple MHT treatments without the need for additional injections, increasing the efficacy compared to a single MHT process (see Figure 26).

### 6.2. Integration of pH-Responsive Polymers

With a pH of ~6.8, the extracellular medium of a tumor is more acidic than that of blood and normal tissues (with a pH of 7.4), and lysosomes are even more acidic (pH~5.0–5.5) [97]. The three-dimensional structure of pH-responsive polymers can expand under basic conditions and collapse under acidic conditions [107]. Consequently, pH-responsive polymers have been investigated by many researchers for possible applications in anticancer drug delivery [117]. For example, diblock copolymers of PEG and 2-(diisopropylamino)ethanol grafted poly(L-aspartic acid) (PEG-PAsp(DIP)) integrated with SPIONS showed significantly higher release of DOX (80% after 2 h and 90% after 24 h) at pH 5 compared to pH 7.4 (<20% after 24 h) [96]. After 2 h of incubation with cells at 37 °C, the drug-loaded nanovesicles were taken up by endocytosis and entrapped inside lysosomes. Importantly, the authors observed that DOX accumulated inside the nuclei, indicating release and migration of DOX from the nanovesicles at lysosomal pH. Moreover, integration with magnetic nanoparticles enabled monitoring of this chemotherapeutic process using MRI.

**Figure 27:**
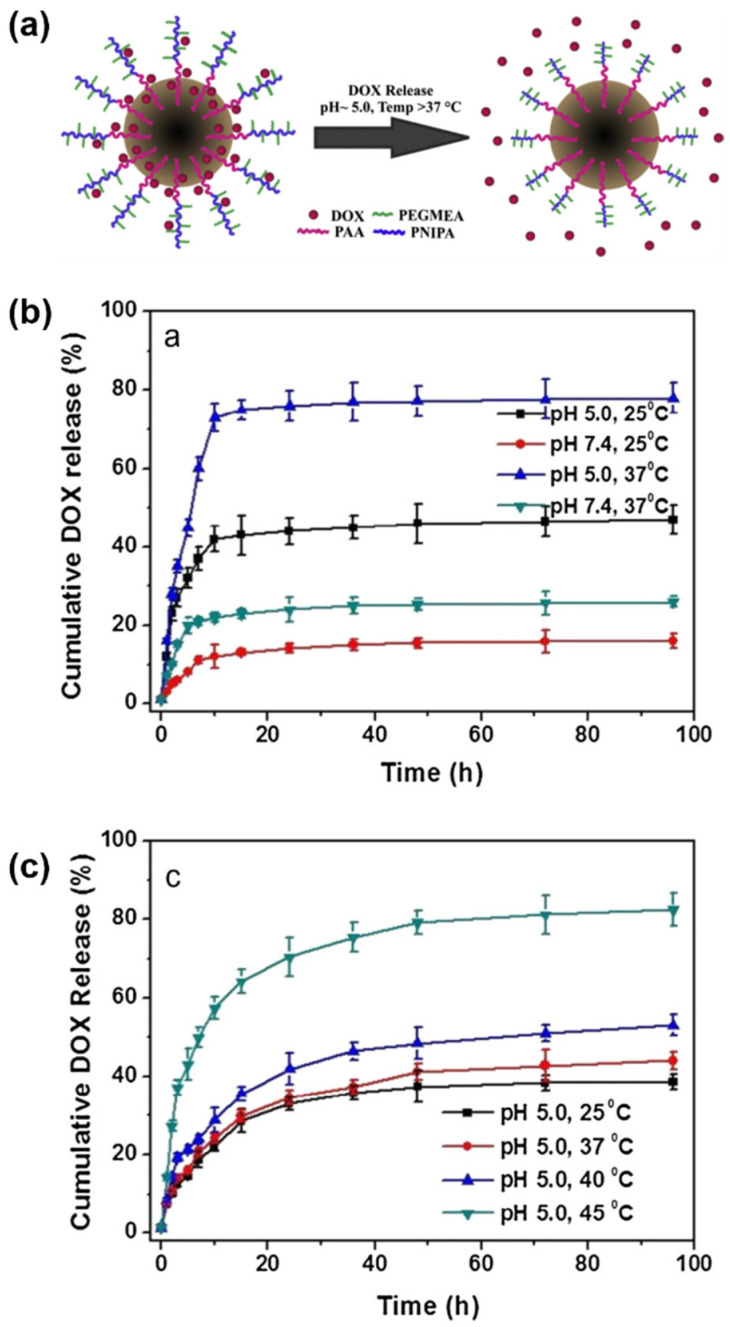
(**a**) Schematic representation of a therapeutic strategy combining LCST-modified thermoresponsive and pH-responsive polymer properties with IONPs. (**b**) DOX release profile from P(NIPAm)_97_-b-(tBA)_51_-DOX-MNPs under different conditions and from (**c**) [P(NIPAm)_85_-r-(PEGMEA)_15_]-b-(PtBA)_51_-DOX-MNPs at pH 5.0 at different temperatures. Reproduced with permission from reference [97]. Copyright 2016 Elsevier.

Recent designs combine both the thermoresponsive and pH-responsive properties of polymers with the MHT and MRI capabilities of IONPs [94,97]. Dutta et al. grafted poly((N-isopropylacrylamide-ran-poly(ethylene glycol) methyl ether acrylate)-block-poly(acrylic acid) (P(NIPAm-r-PEGMEA)-b-PAAc) polymers onto IONPs and investigated the effects of polymer composition, temperature, and pH on DOX release [97]. The results showed a much higher drug release at pH 5.0 than at pH 7.4, while an increase in the number of PEGMEA units restricted drug release at temperatures of 37 and 40 °C (see Figure 27). Thus, temperature-controlled release of DOX was achieved at 45 °C, preferentially in lysosomes of cancer cells at pH 5.0. Overall, the above examples demonstrate that integration of magnetic NPs and stimuli-responsive polymers allows the development of new nanotechnology-based therapeutic strategies. These platforms might contribute to future controlled drug-release systems, novel therapeutics, and new contrast agents in which the drugs or agents can be released by exposure to an oscillating magnetic field, while the therapeutic results can be synergistically monitored and diagnosed using MRI techniques.

## 7. Integration of Multiple Conjugates with Magnetic Iron Oxide Nanoparticles

In addition to NP-based systems that integrate magnetic iron oxide NPs with one of the five conjugate types mentioned above, numerous studies have developed platforms for biomedical applications by combining magnetic IONs with multiple conjugates, such as an organic dye and a biomolecule or a biomolecule with a stimuli-responsive polymer. These systems can offer various advantages due to their ability to perform several tasks in parallel, such as cancer cell targeting, dual-mode imaging, drug delivery, and therapy [118,119,120,121,122]. Table 6 provides a summary of multiconjugate integrated magnetic iron oxide NP-based platforms for biomedical applications.

**Figure 28:**
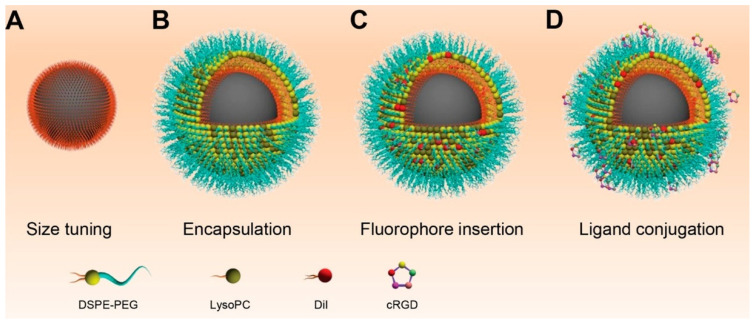
Illustrated design of lipid-encapsulated organic dye-doped polymer-coated iron oxide MNPs for dual-MRI and FI. Reproduced with permission from reference [15]. Copyright 2020 American Chemical Society.

Very recently, the Bao group developed lipid-encapsulated Fe_3_O_4_ nanoparticles as a contrast agent in multimodal MRI/FI [15]. Specifically, magnetite NPs with a well-controlled size distribution were synthesized via thermodecomposition before being coated with copolymers of phospholipids and phospholipid-PEG to generate water-soluble MNPs. Then, these polymer-coated MNPs were integrated with an organic dye dialkylcarbocyanine, such as DiO, DiI, DiD, or DiR, via hydrophobic interactions of the organic dyes with the lipid layer of the shell of the MNPs. Finally, nanoprobes with desired sizes and optical and magnetic properties were achieved by conjugation of the bioactive ligands cyclic RGD or cyclic RAD peptides to the nanoprobes [15]. Figure 28 presents an illustration of the design of these lipid-encapsulated organic dye-doped iron oxide MNPs, which exhibited high stability in biomedical media and good biocompatibility. Moreover, the conjugated peptides greatly increased the uptake of these nanoprobes by cells. In vitro and in vivo experiments revealed good fluorescence signals and MRI contrast, demonstrating potential application of these MNPs in biomedical fields [15].

**Figure 29:**
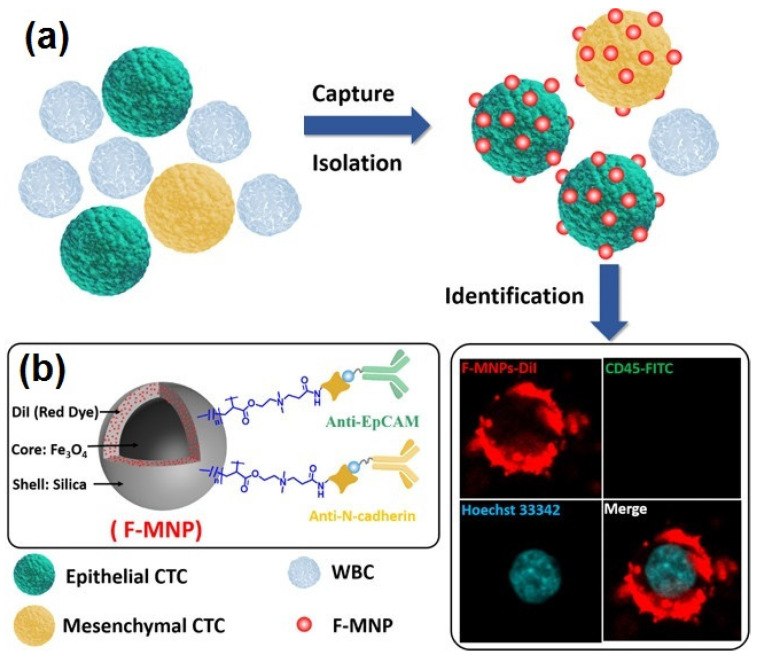
(**a**) Capture and identification of circulating tumor cells (CTCs) using fluorescent magnetic nanoparticles (F-MNPs) and (**b**) the structure of the antibody-functionalized modified F-MNPs. Adapted with permission from reference [133]. Copyright 2019 American Chemical Society.

In another investigation, Wang et al. reported the fabrication of anti-epithelial cell adhesion molecule (anti-EpCAM) and anti-N-cadherin antibodies onto fluorescent Fe_3_O_4_ magnetic nanoparticles (F-MNPs) to detect epithelial circulating tumor cells (CTCs), which indicate early cancer development [133]. Due to the characteristic recognition of the antibodies, the dual-antibody functionalized F-MNPs can selectively bind to CTCs before being isolated using an external magnetic field and identified under a fluorescence microscope, as shown in Figure 29a. The magnetite magnetic core was encapsulated with a fluorescent dye DiI-decorated silica shell before being modified with poly(carboxybetaine methacrylate) (pCBMA), streptavidin, and antibodies, as illustrated in Figure 29b.

**Figure 30:**
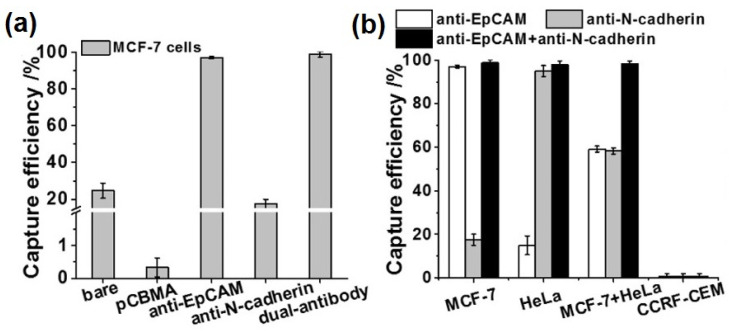
Comparison of the capture efficiency of F-MNPs under various modification conditions (**a**) for MCF-7 cells and (**b**) of anti-EpCAM-modified, anti-N-cadherin-modified, dual-antibody-modified F-MNPs for different cells. Adapted with permission from reference [133]. Copyright 2019 American Chemical Society.

The authors evaluated the MCF-7 human breast cancer cell line capture efficiency of F-MNPs at various modification steps. The results shown in Figure 30a suggest the highest capture efficiency of 98.8% for F-MNPs functionalized with both anti-EpCAM and anti-N-cadherin antibodies. The modified magnetic nanoparticles were also evaluated for their recognition performance, with MCF-7, HeLa, and CCRF-CEM cells used as cell models for epithelial, mesenchymal CTCs, and blood cells, respectively. The dual-antibody-modified F-MNPs showed higher capture efficiencies for both MCF-7 and HeLa cells than single-antibody-modified nanoparticles, as shown in Figure 30b. Additionally, the selective CTC identification and capture capability of the modified F-MNPs was shown by the near-zero capture efficiency of the human T lymphocytic leukemia cell line CCRF-CEM, suggesting that the antibody-modified magnetic nanoparticles have potential for efficient early cancer detection. In a separate work, White et al. fabricated magnetic-plasmonic ultrasmall superparamagnetic iron oxide (USPIO)-gold hybrid nanoparticles with anti-MG1 antibodies for targeted photothermal ablation of colorectal liver metastases [134]. Moreover, Dong and coworkers prepared fluorescent magnetic mesoporous silica nanoparticles (M-MSNs) conjugated with fluorescein isothiocyanate (FITC) before modifying them with EpCAM antibody for efficient detection of circulating tumor cells (CTCs) [135].

Wang et al. developed improved magnetic-core@dual QD-shell nanoparticles (Fe_3_O_4_@DQDs) as multifunctional fluorescent labels for fluorescence lateral flow detection of bacteria [136]. In this study, mercapto-propionic acid-functionalized QDs (CdSe/ZnS-MPA) were chosen as dual QDs due to their outstanding and stable fluorescence properties. Interestingly, the QD-adhering step was carried out two times to obtain the final targeted Fe_3_O_4_@DQDs. Compared to Fe_3_O_4_@QDs, the Fe_3_O_4_@DQDs had a higher number of QDs, leading to fluorescence enhancement of the whole nanostructure. For ultrasensitive bacteria detection, the combination of the magnetic property of Fe_3_O_4_ and the high fluorescence of DQDs led to a highly sensitive bacteria detection LFA strip.

**Figure 31:**
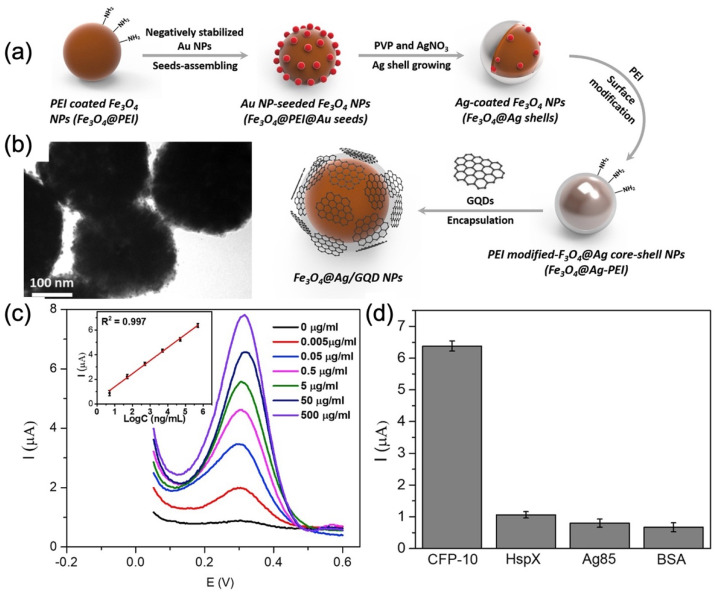
(**a**) Preparation steps for the Fe_3_O_4_@Ag/GQD NP probe. (**b**) TEM image of Fe_3_O_4_@Ag/GQD NPs. (**c**) Peak value of the electrochemical biosensor for CFP-10 detection at concentrations ranging from 0 to 500 μg/mL. (**d**) DPV responses of the immune sensors to CFP-10 (0.5 mg/mL), HspX protein of Mtb (1.8 mg/mL), Ag85 (3.57 mg/mL), and 2% BSA (3.57 mg/mL). Adapted with permission from reference [140]. Copyright 2018 Elsevier.

Recently, the Lee group reported a nanotriplex particle Fe_3_O_4_@Ag/graphene quantum dot (GQD) as an electrochemical immunosensor for tuberculosis [140]. Because of the Fe_3_O_4_ core, the nanomaterial had excellent magnetic properties and good water solubility, as well as a large surface area due to its nanomorphology. Ag NPs were incorporated into the nanomaterial to increase conductivity. For electrochemical immunosensor applications, graphene quantum dots (GQDs) were integrated because they are great electron donors and acceptors, which is rationalized by the large surface area and the presence of multiple types of functional groups on the QD surface. The preparation of Fe_3_O_4_@Ag/GQDs is presented in Figure 31a, and Figure 31b shows TEM images of the nanotriplex, revealing a diameter of ~270 nm. As shown in Figure 31c, the differential pulse voltammetry (DPV) peak was enhanced when the concentration of the analyte culture filtrate protein CFP-10 increased during immunocomplex formation. The system showed a robust performance and high selectivity for CFP-10 in the presence of antigen 85 complexes (Ag85), HspX protein of Mtb (16 kDa), and bovine serum albumin (BSA), as shown in Figure 31d.

Irudayaraj and coworkers fabricated a novel “nanopearl-necklace” structure that consists of a single gold nanorod (Au_rod_) decorated with multiple “pearls” of 15 nm Fe_3_O_4_ magnetic NPs for use in simultaneous targeting, bimodal imaging, and photothermal ablation of cancer cells [121]. In vitro assays of this system revealed that Au_rod_-(Fe_3_O_4_) exhibited a stronger MR signal than bare Fe_3_O_4_ at an equivalent iron concentration due to the magnetic coupling between the Fe_3_O_4_ particles assembled on the Au_rod_ core. Conjugates derived from covalent attachment of Herceptin to the Au_rod_-(Fe_3_O_4_) nanocomplexes were observed to bind specifically to cancer cell surfaces and were internalized into the cytoplasm, which was verified by MR and transmission electron microscopy (TEM) images. An additional advantage of these nanocomposites is that the inherent fluorescence of the Au_rod_ component allows FI analysis. These studies indicate that such nanocomposites can potentially serve as contrast agents for both MRI and FI modalities. Furthermore, the use of these Herceptin-conjugated Au_rod_-(Fe_3_O_4_) NPs as therapeutic agents was also demonstrated. Once the Herceptin-conjugated Au_rod_-(Fe_3_O_4_) NPs were ingested and allowed to accumulate in the internal vesicles and cytoplasm of cancer cells, the NPs were irradiated with a 785 nm NIR laser. The absorbed energy heated the Au_rod_, allowing local destruction of cancer cells and illustrating efficient photothermal ablation.

**Figure 32:**
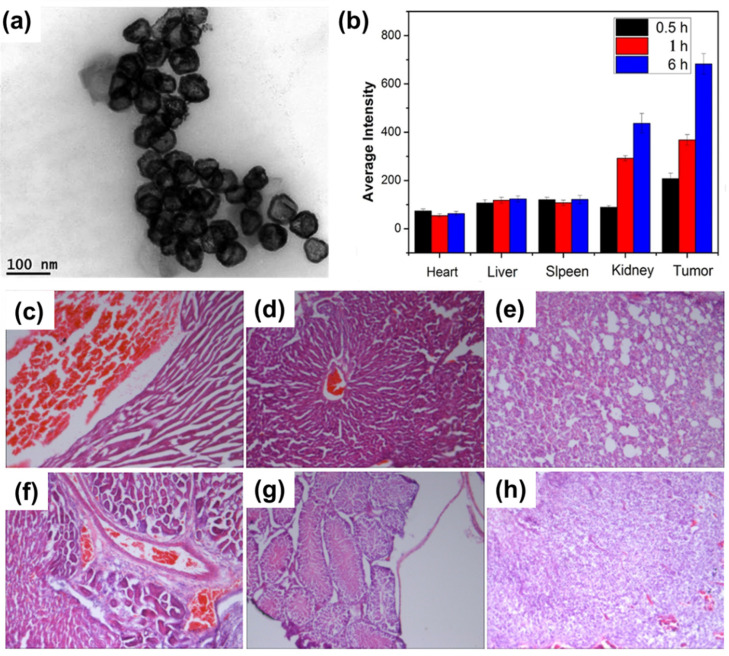
(**a**) TEM image of F-AuNC@Fe_3_O_4_. (**b**) Average Hounsfield unit (HU) intensity in the heart, liver, spleen, kidney, and tumor after intravenous injection. H&E staining of organ sections: (**c**) heart, (**d**) liver, (**e**) lung, (**f**) kidney, (**g**) bladder, and (**h**) tumors harvested from mice at 6 h postinjection. Adapted with permission from [141].

Gold nanocages functionalized with Fe_3_O_4_ nanoparticles (F-AuNC@Fe_3_O_4_) have also been studied for multimodal imaging of tumors (Figure 32) [141]. In this study, nanocomposites were used as multimodal contrast agents for MRI/computed tomography (CT) multimodal imaging. The aforementioned nanoparticles were conjugated with folic acid to selectively bind with targeted folate receptor-overexpressing cancer cells. The results from an in vivo CT imaging study showed that F-AuNC@Fe_3_O_4_ can enhance CT imaging in the circulatory system. As shown in Figure 32b, the average signal intensity in kidney and tumor tissues was enhanced from 0.5 h to 6 h, while the signal in other organs showed fluctuation. Additionally, to study the biocompatibility of F-AuNC@Fe_3_O_4_ nanoparticles, the nanoparticles were tested via a pathological assay (H&E staining). As shown in Figure 32c–h, the results demonstrated that F-AuNC@Fe_3_O_4_ nanoparticles were present in the kidney, bladder, and tumor at 6 h after injection. However, the nanoparticles were absent in the liver and heart after the same period of time. Additionally, no morphological changes were observed in the organs, suggesting that the nanoparticles were biocompatible.

Separate studies have described the fabrication of nanocomplexes composed of spherical gold nanoshell/silica core structures that were subsequently coated with 10 nm magnetic NPs and an outer SiO_2_ layer doped with an NIR dye [142]. In this geometric structure, the gold nanoshell absorbs light in NIR regions and can also significantly enhance the fluorescence of the adjacent NIR dye molecules, thus significantly improving the resolution of fluorescence images. Furthermore, incorporation of magnetic NPs on the gold nanoshell surfaces led to increased magnetic interactions among the particles, thereby enhancing the relaxivity and improving the MR signals. Additionally, the silica outer layer can be readily modified with biomolecular entities for cell or protein targeting. Furthermore, the ability to absorb NIR light and produce heat from the gold nanoshell component enables these hybrid NPs to be used for photothermal ablation. Consequently, these integrated properties allowed the use of this platform in multiple diagnostic and therapeutic modalities.

Surface-enhanced Raman spectroscopy (SERS)-encoded MNPs and AgNP-embedded SiO_2_-coated Fe_3_O_4_ NPs (M-SERS) were reported as multifunctional materials for cancer cell targeting and separation [143]. In this study, M-SERS nanoparticles were functionalized with various types of thiol-group-containing organic compounds to demonstrate the enhancement of Raman scattering. Additionally, due to the presence of selected aromatic compounds, the aforementioned nanoparticles exhibited strong SERS signals. To demonstrate the magnetic properties of the M-SERS nanoparticles, an external magnetic force was applied to the nanoparticles after conjugation with antibodies and targeted molecules. The targeted cells with the M-SERS nanoparticles moved toward the magnet under an external magnetic field. These results confirmed that the M-SERS nanoparticles are promising materials for detection and separation of biomolecules.

In another study, Li et al. used a thermally sensitive and hepatic tumor-cell-targeting peptide-A54-modified polymer, A54-poly(ethylene glycol)-g-poly(acrylamide-co-acrylonitrile) (A54-PEG-g-p(AAm-co-AN)), assembled into 80 nm sized micelles, allowing DOX drug transport and release and augmenting microwave hyperthermia at 43 °C [144]. Instead of LCST, the operation of these polymers was centered around their upper critical solution temperature (UCST). Other thermally responsive polymers include those with glass transition temperatures (T_g_) that coincide with typical MHT temperatures. For example, IONP-embedded DOX-containing carboxylic polypyrrole systems soften above their T_g_ of 44 °C, leading to the release of DOX [116]. These NPs were further modified with PEG functionalized with folic acid (FA), and FA specifically binds to folate receptors overexpressed in cancer cells. Tumor temperatures of 44 °C were reached within 7 min of AMF exposure, killing cancer cells throughout the entire tumor.

**Figure 33:**
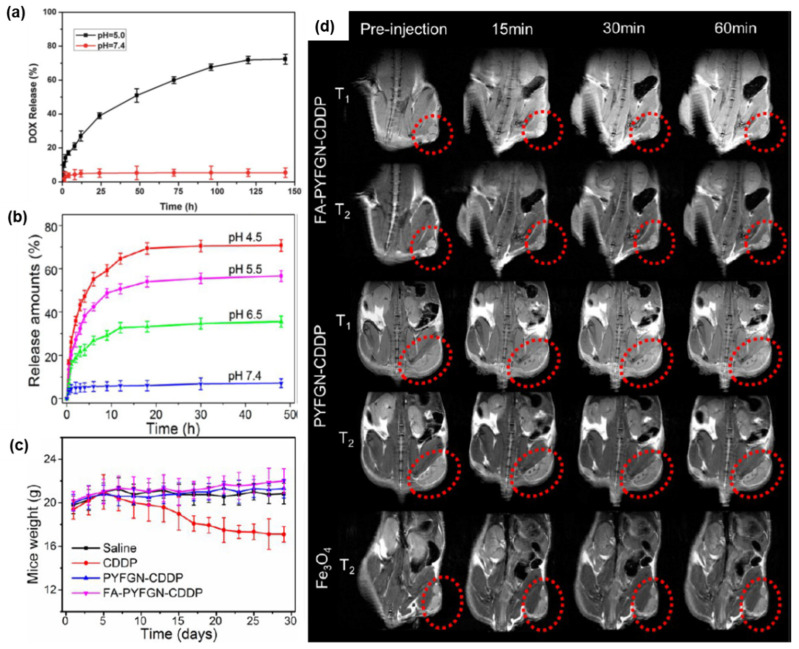
(**a**) Release of DOX in vitro from drug-loaded pH-responsive PEG-PBLA-coated hollow SiO_2_ NPs with SPIONs at different pH values at 37 °C. Reproduced with permission from reference [146]. Copyright 2012 Royal Society of Chemistry. (**b**) Cisplatin release from Fe_3_O_4_@Gd_2_O_3_ yolk-shell NPs functionalized with PEG and FA (FA-PYFGN-CDDP) under different pH conditions. (**c**) Body weight changes after tumor-bearing nude mice were treated with free cisplatin (CDDP) and cisplatin-loaded Fe_3_O_4_@Gd_2_O_3_ yolk-shell NPs functionalized with PEG (PYFGN-CDDP) or FA (FA-PYFGN-CDDP) via tail vein injection. (**d**) Gd_2_O_3_-weighted (T1) and Fe_3_O_4_-weighted (T2) MR images of mouse tumors acquired preinjection and postinjection of FA-PYFGN-CDDP and PYFGN-CDDP. Tumors are marked by red dashed circles. Reproduced with permission from reference [122]. Copyright 2017 American Chemical Society.

As mentioned earlier, the inclusion of folic acid (FA) moieties can improve tumor-specific delivery of pH-responsive integrated nanocarriers via attachment to folate receptors overexpressed in cancer cells. Yang et al. designed triblock polymers with varying *M_w_* consisting of (folate (FA) or methoxy)-poly(ethylene glycol) (*Mw*: 5000)-poly(glutamate hydrozone doxorubicin)-poly(ethylene glycol) (*M_w_*: 2000)-acrylate (i.e., R (FA or methoxy)-PEG_114_-P(Glu-Hyd-DOX)-PEG_46_-acrylate) [145]. The longer PEG chains containing FA segregated toward the outer layer of the vesicles and facilitated targeted endocytic delivery, while the shorter PEG block segregated to the inner region of the vesicles and facilitated cross-linking and enhanced the in vivo stability of the nanovesicles. In another design, hollow SiO_2_ NPs integrated with hydrophobic polymers containing folic acid and SPIONs demonstrated drug release in an acidic environment in cancer cells; 70% of DOX was released at pH 5.0, whereas less than 5% was released in a neutral environment after 150 h (see Figure 33a) [146]. The hollow SiO_2_ core enabled high drug loading efficiency, and targeted delivery was tracked via MRI-active SPIONs. The MRI contrast of Fe-based nanoparticles can be further enhanced by combination with Gd-based compounds, as shown by Sun et al. with their Fe_3_O_4_@Gd_2_O_3_ yolk-shell NPs functionalized with PEG and FA [122]. The yolk-shell design with a porous Gd_2_O_3_ shell improved MRI contrast, increased drug loading, and enabled pH-induced release of the anticancer drug cisplatin inside tumor cells (see Figure 33b–d). This targeted drug delivery nanosystem also demonstrated a marked reduction in damage to vital organs compared with that observed with free cisplatin.

**Figure 34:**
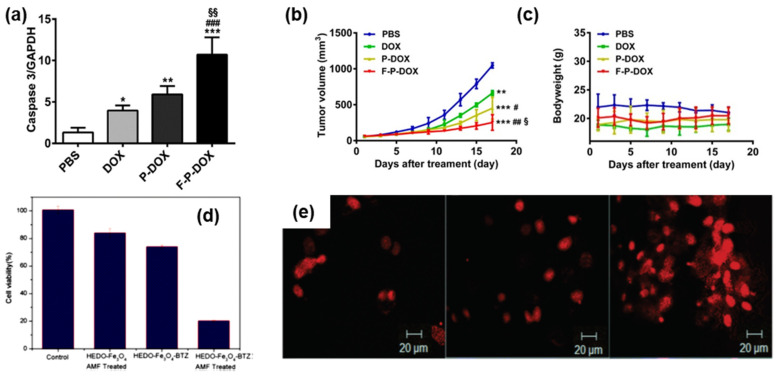
(**a**) The mRNA expression of Caspase 3, an apoptosis-associated gene, was examined via RT–PCR upon exposure to PBS buffer, free DOX, or IONPs integrated with poly(*β*-aminoester) (P-DOX) and folate (F-P-DOX). (**b**) Tumor growth curves and (**c**) body weight curves demonstrating the improved antitumor efficacy of F−P-DOX in a GC xenograft model, without obvious adverse effects. Each experiment was conducted in triplicate. * *p* < 0.05, ** *p* < 0.01, *** *p* < 0.001 vs PBS group; # *p* < 0.05, ## *p* < 0.01, ### *p* < 0.001 vs DOX group; § *p* < 0.05, §§ *p* < 0.01 vs P- DOX group. Reproduced with permission from reference [147]. Copyright 2015 American Chemical Society. (**d**) The in vitro anticancer effects of p(HEMA-co-DMA)-integrated IONPs loaded with BTZ (HEDO–Fe_3_O_4_–BTZ) on SCC7 cell lines. (**e**) Magic RedTM assay showing the apoptosis-inducing effect (red fluorescence) with hyperthermia alone (HEDO–Fe_3_O_4_, AMF on), chemotherapy alone (HEDO–Fe_3_O_4_–BTZ), and combined application of hyperthermia and chemotherapy (HEDO–Fe_3_O_4_–BTZ, AMF on). Adapted with permission from reference [149]. Copyright 2015 Royal Society of Chemistry.

Folate-conjugated, pH-sensitive poly(*β*-aminoester) self-assembled micelles with hydrophobic oleic acid-modified IONPs delivering DOX were shown to facilitate the treatment of advanced gastric cancer via apoptosis of cancerous cells [147]. As seen in Figure 34a–c, once again, the folate conjugates showed much greater suppression of tumor growth without affecting the overall body weight and can be safely monitored via MRI. Yang et al. also used methyloxy-poly(ethylene glycol)-block-poly[dopamine-2-(dibutylamino) ethylamine-L-glutamate] (mPEG-b-P(DPA-DE)LG) micelles [148]. Dopamine has a high affinity for IONPs, enhancing stability at physiological pH, while the mPEG moiety enhances dispersion in aqueous media. These nanoparticles acted as pH-sensitive MRI probes, releasing IONPs at an acidic pH. Sasikala et al. used a unique copolymer with dopamine, poly(2-hydroxyethyl methacrylate codopamine methacrylamide) p(HEMA-co-DMA) to surface functionalize IONPs, taking advantage of dopamine’s affinity for IONPs [149]. The catechol groups in dopamine were also exploited to conjugate the borate-containing anticancer drug bortezomib (BTZ). In this study, the researchers exploited the MHT capabilities of SPIONs along with pH-sensitive drug release for synergistic thermochemotherapy. The synergistic effect of BTZ and MHT therapy increased the apoptosis of cancer cells by nearly 4-fold compared to BTZ release or MHT alone (Figure 34d,e).

## 8. Summary and Perspectives

This review examines a variety of current composite magnetic IONPs that have been integrated with different classes of organic and inorganic materials for biomedical applications. Research efforts have focused on integrating iron oxide MNPs and organic dyes into single platforms for use as bimodal imaging agents for both in vitro and in vivo imaging and to produce multifunctional platforms that simultaneously perform several tasks in parallel, such as photodynamic therapy and dual-mode imaging for biomolecule detection. Importantly, these platforms can provide better diagnostic information and a more complementary dataset due to their dual-diagnostic MRI/FI modalities than the corresponding isolated imaging techniques. Due to their high biocompatibility, low cytotoxicity, and low risk of immune rejection, various biomolecules, such as aptamers, antibodies, and even stem cells, can be integrated with magnetic IONPs to develop highly targeted techniques for diagnosis, therapy, and theranostics for many notorious diseases, and the results have been promising.

Quantum dots (QDs), such as CdSe, ZnS, and CdS, and carbon dots (CDs), are recognized for their unique optical properties, such as resistance to photobleaching, high molar extinction coefficient, and tunable emission ranges. Consequently, QDs are often incorporated with magnetic NPs to produce composite NPs that exhibit both fluorescence and magnetism, and the effects of these composite NPs can be examined in vitro before progressing to in vivo tests. However, the use of magnetic-QD platforms in biomedical applications is limited due to the inherent toxicity of many current QDs. Thus, more biocompatible QDs are needed for broader use of magnetic-QD platforms in biomedical applications. In addition to QDs, combining noble metal NPs and magnetic NPs into one platform offers multiple diagnostic and therapeutic modalities simultaneously, which can improve the accuracy and clarity of diagnostic images while reducing time and expense. Magnetic-noble metal NP-based platforms combine the magnetic properties of IONPs with remarkable optical properties that arise from surface plasmon resonances, which can be controlled from the ultraviolet to the near-infrared (NIR) regions of the electronic spectrum by optimizing the size, composition, shape, and topology of noble metal NPs.

Moreover, integrating magnetic nanoparticles with polymers allows for fabrication of multifunctional systems. Encapsulation and cross-linking with polymers provide improved stability, circulation, biocompatibility, and pH-sensitive magnetic resonance imaging. An alternating magnetic field can be applied to heat magnetic nanoparticles in vivo, which can be used to stimulate thermosensitive and pH-sensitive polymers for targeted drug delivery and chemotherapy. These advanced anticancer drug delivery capabilities can also be coupled with magnetic hyperthermia therapy to kill cancer cells more effectively. These chemotherapy and magnetic hyperthermia therapy processes can also be simultaneously monitored by utilizing the magnetic resonance imaging capabilities of magnetic nanoparticle–polymer conjugates. Thus, coupling all of these unique properties of polymers and magnetic nanoparticles enables the design and fabrication of more effective biomedical materials. In addition, appropriate engineering and integration of magnetic IONPs with multiple conjugates, such as an organic dye and a biomolecule or a biomolecule with a stimuli-responsive polymer, can generate multifunctional nanoplatforms that can perform multiple tasks simultaneously and be used in a broad range of biomedical fields.

## Figures and Tables

**Table 1:** Organic dye-integrated magnetic nanoparticles for biomedical applications.

NP	Conjugate	Morphology	Final Size (nm)	Applications	Ref.
IONPs@ DPSE-PEG	DiO, or DiI, or DiD, or DiR	Spherical	24–46	Multimodal MRI and FI	[15]
SPIONs	Ce6	Spherical	92	Theranostic agent for dual-mode imaging and photodynamic therapy	[16]
SPIONs@ DSPE-PEG	ICG	Spherical (core-shell)	23	Tumor MR and fluorescence imaging and drug delivery for DOX	[17]
Fe_3_O_4_@SiO_2_-CMCS	Cy5	Spherical (core-shell)	51	Bioimaging	[18]
Fe_3_O_4_@poly(HFMA-co-VBK)-g-PEG	VBK	Spherical (core-shell)	146	Magnetic resonance and optical imaging	[19]
GO-PAMAM-Fe_3_O_4_	Cy5	Irregular	-	Cellular imaging	[20]
Fe_3_O_4_-P(PEGMA)	FITC	Spherical	36	Bioimaging	[21]
Fe_3_O_4_	Ce6	Spherical	15–25	Dual-mode NIR fluorescence imaging and MRI of gastric cancer and photodynamic therapy (PDT)	[22]
mSiO_2_-Fe_3_O_4_-PEG	RITC or FITC	Spherical	93	Enhanced MRI, FI, and drug delivery for DOX	[23]
Fe_3_O_4_@mSiO_2_-PEG	FITC or RITC	Spherical (core-shell)	45–105	MRI and FI	[24]
IONPs@PSSS-PAH	RhB	-	-	Bioimaging	[25]
γ-Fe_2_O_3_@CS	FITC	-	14	Cellular imaging	[26]
SPIONs@poly(TMSMA-r-PEGMA-r-NAS)	Cy5.5	Core-shell	~26	Dual-mode MRI and optical imaging	[27]
SPIONs@SiO_2_	FITC	Spherical (core-shell)	50	MRI for human stem cell labeling	[28]
γ-Fe_2_O_3_	RhB or fluorescein diacetate maleimide	~	~30	Cellular imaging	[29]
IONPs-cross-linked dextran	Cy5.5	~	~	Preoperative MRI and intraoperative optical probe	[30]
γ-Fe_2_O_3_	CR or RITC	Spherical	14–15	Multimodal imaging agents for amyloid-β fibril detection and removal	[31]

**Abbreviations:** IONPs: iron oxide nanoparticles; SPIONs: superparamagnetic iron oxide nanoparticles; DPSE-PEG: 1,2-distearoyl-sn-glycero-3-phosphoethanolamine-*N*-[methoxy(polyethylene glycol)]; MRI: magnetic resonance imaging; FI: fluorescence imaging; Ce6: chlorin e6; ICG: indocyanine green; DOX: doxorubicin; Cy5: squarylium indocyanine dye; HFMA: 2,2,3,4,4,4-hexafluorobutyl methacrylate; VBK: 9-(4-vinylbenzyl)-9H-carbazole; PEG: poly(ethylene glycol); P(PEGMA): poly(poly(ethyleneglycol)monomethacrylate); FITC: fluorescein isothiocyanate; NIR: near-infrared; PDT: photodynamic therapy; RITC: rhodamine B isothiocyanate; mSiO_2_: mesoporous silica; PSSS: polysodium-4-styrene sulfonate; PAH: poly(allylamine hydrochloride); RhB: rhodamine B; Cy5.5: cyanine-5.5; TMSMA: 3-(trimethoxysilyl)propyl methacrylate; NAS: *N*-acryloxysuccinimide; CR: Congo red.

**Table 2:** Biomolecule-functionalized magnetic iron oxide nanoparticles for biomedical applications.

NP	Conjugate	Morphology	Final Size (nm)	Applications	Ref.
IONPs	GRAA33 aptamer	Spherical	68 ± 4	Remove prefibrillar amyloid aggregates	[34]
IONPs	Genomic DNA	Irregular	NA	Capture DOX, 98%	[35]
SPIONs	AS-14, AS-42	Irregular	~20	Cancer treatment	[36]
IONPs	DNA	Spherical	50 ± 5	Detect and regulate mRNA, 99%	[37]
IONPs	ssDNA	Spherical	200–400	AFP capture for pregnancy test, 87%	[38]
γ-Fe_2_O_3_	DNA	Irregular	NA	Thrombin detection, limit of 97 pM	[39]
IONPs	Anti-K2 antibody	Spheroidal	17–25	JWH-073 detection	[40]
SPIONs	mAb	NA	NA	Cancer drug delivery	[41]
IONPs	Anti-ferritin antibody	Spherical	~50	Alzheimer’s disease early diagnosis	[42]
IONPs	Anti-fFN antibody	Spherical	37–63	Preterm birth diagnosis	[43]
USPIO	CKAAKN peptide	Spheroidal	~60	Enhanced MRI contrast agent	[44]
IONPs	Biotin, folic acid	Spherical	~70	Anticancer drug carrier	[45]
IONPs	Lipidoid	Spherical	65 ± 5	MRI-trackable immunotherapy	[46]
IONPs@Al_2_O_3_	Peptide	Irregular		Staphylococcus aureus pathogen screening	[47]

**Abbreviations:** IONP: iron oxide nanoparticle; SPIONs: superparamagnetic iron oxide nanoparticles; mAb: anti-Met/HGFR-positive; NA: not available; USPIO: ultrasmall superparamagnetic iron oxide; anti-fFN antibody: anti-fibronectin antibody; DOX: doxorubicin; CTC: circulating tumor cell; EpCAM: epithelial cell adhesion molecule; MRI: magnetic resonance imaging.

**Table 3:** Integration of QDs and magnetic iron oxide NPs for biomedical applications.

NP	Conjugate	Morphology	Final Size (nm)	Applications	Ref.
Fe_3_O_4_@CDs		Sphere	100	Dual-modal imaging, near-IR light-responsive drug carrier, and photothermal therapy	[60]
Fe_3_O_4_/SiO_2_/graphene-CdTe QDs/chitosan nanocomposites	Chitosan	Sphere	177	Drug delivery	[61]
Fe_3_O_4_@SiO_2_@ CdSe-CdS-ZnS QDs		Sphere	75	Potential use in magnetic field-assisted cell separation	[62]
QD–Pt(II)–6G–IONP	(diamine)PtCl_2_ complexes	Sphere	38	Potential use in combined bioimaging systems such as FMT and MRI	[63]
Fe_3_O_4_/graphene–QDs@gelatin microspheres	Gelatin	Sphere	460	Drug delivery	[64]

**Abbreviations:** CDs: carbon dots; IONP: iron oxide nanoparticle; MRI: magnetic resonance imaging; FMT: fluorescence molecular tomography.

**Table 4:** Integration of noble metal NPs and magnetic NPs for biomedical applications.

NP	Morphology	Final Size (nm)	Applications	Ref.
Fe_3_O_4_@Au	Nanosphere (core–shell)	20	Photothermal therapy	[75]
	Nanospikes	48–52	Theragnostic agent	[76]
Fe_3_O_4_@Au-mPEG-PEI	Nanosphere (core–shell)	263	MR/CT dual-mode imaging	[77]
Fe_3_O_4_@SiO_2_@ GNSs-PEG	Nanosphere	~100	MR/CT dual imaging and photothermal therapy	[78]
Fe_3_O_4_@Ag-PAA	Nanosphere (core–shell)	~10	Photothermal therapy	[79]
Fe_3_O_4_-Ag-PAA	Nanosphere (heteromer)	~10	Photothermal therapy	[79]
SPION-Ag	Nanosphere (core–shell)	~30	Antibacterial	[80]
SPION-Au	Nanosphere (core–shell)	~30	Antibacterial	[80]
SPION-Au/Ag	Nanosphere (core–shell)	~40	Antibacterial	[80]

**Abbreviations:** AuNC: gold nanocube; AuNS: gold nanoshell; PEG: polyethylene glycol; MR: magnetic resonance; CT: computed tomography; mPEG-PEI: polyethyleneimine-poly(ethylene glycol) monomethyl ether; PAA: poly(acrylic acid); SPION: superparamagnetic iron oxide nanoparticle.

**Table 5:** Stimuli-responsive polymer-integrated magnetic NPs for biomedical applications.

NP	Conjugate	Morphology	Final Size (nm)	Applications	Ref.
IONPs	PLGA	Nonspherical	80–90	AMF-triggered DDS (LCST)	[84]
SPIONs@ carbon	PNIPAm-MBAm	Spherical (core–shell)	280	Inductive magnetic heating-triggered DDS (LCST) for 5-Fluoruoracil	[87]
IONPs	PNIPAm-co-AAc	Irregular	14–43	AMF-triggered MHT and DDS (LCST) for DOX	[88,89]
IONPs@SiO_2_	PNIPAm-co-PVNP	Yolk-shell	~165	NIR-triggered DDS (LCST) for DOX	[90]
Fe_3_O_4_ and γ-Fe_2_O_3_	PNIPAm-co-HAAm	MNPs embedded in polymer nanofiber	~350	AMF-triggered MHT and DDS (LCST) for DOX	[91]
IONPs	PNIPAm-co-PEGMEA	Cubic	19–22	AMF-triggered DDS (LCST) for DOX	[92]
SPIONs	PPZ	Spherical	153	AMF-triggered MHT, and MRI	[93]
Fe_3_O_4_	PNIPAm, PEG, dopamine	Spherical	200	AMF-triggered MHT and DDS (LCST) for DOX, and MRI	[94]
IONPs	PEO-b-PPO-b-PEO, gelatin	Spherical	28–36	AMF-triggered DDS (LCST)	[95]
SPIONs	PEG-b- PAsp(DIP)	Spherical nanovesicles	~200	DDS (pH) for DOX, and MRI	[96]
IONPs	[PNIPAm-r(PEGMEA)]-b-PAA	Irregular	23–27	AMF-triggered MHT and DDS (LCST + pH) for DOX, and MRI	[97]

**Abbreviations:** IONPs: iron oxide nanoparticles; SPIONs: superparamagnetic iron oxide nanoparticles; MNPs: magnetic nanoparticles; AMF: alternating magnetic field; MHT: magnetic hyperthermia therapy; MRI: magnetic resonance imaging; DDS: drug delivery system; NIR: near-IR light; LCST: lower critical solution temperature; UCST: upper critical solution temperature; T_g_: glass transition temperature; T_c_: Curie temperature; PLGA: poly(lactic-co-glycolic acid); PNIPAm: poly(N-isopropyl acrylamide); MBAm: *N*,*N*’-methylenebisacrylamide; AAc: acrylic acid; PNVP: poly(1-vinyl-2-pyrrolidone); PEGMEA: polyethylene glycol methyl acrylate); PEG: polyethylene glycol; PPyCOOH: carboxylic polypyrole; PPZ: poly(organophosphazene); P(EO-co-PO)-b-PLL: poly(ethyleneoxide-co-propyleneoxide)-b-poly(L-lysine); PAsp(DIP): poly(2-(diisopropylamino)ethyl aspartate; FA: folic acid; Glu-Hyd: glutamate hydrozone; mPEG-b-P(DPA-DE)LG: methyloxy-poly(ethylene glycol)-block-poly[dopamine-2-(dibutylamino) ethylamine-L-glutamate]; p(HEMA-co-DMA): poly(2-hydroxyethyl methacrylateco-dopamine methacrylamide); DOX: doxorubicin; BTZ: bortezomib.

**Table 6:** Multiconjugate integrated magnetic nanoparticles for biomedical applications.

NP	Conjugates	Morphology	Final Size (nm)	Applications	Ref.
IONPs@DPSE-mPEG	DiI and Cyclic RGD or Cyclic RAD	Spherical (core–shell)	24–46	Multimodal tumor-targeting MRI and FI	[15]
SPION@PEG	Cy5.5 and gH625	-	98	Multimodal tumor-targeting MRI, FI, and hyperthermia	[118]
Gelatin@IONPs	Cy7 and HSA-DVS-FGF2	Spherical (core–shell)	>103	Controlled release of growth factors for augmentation of human mesenchymal stem growth and differentiation	[123]
Fe_3_O_4_@SiO_2_@ p(HEMA)	FITC, FA, and p(NIPAAM-co-AA)	Spherical (core–shell)	~	Dual thermoresponsive and pH-sensitive drug delivery for DOX	[124]
Fe_3_O_4_@PEI@ PEG	Cy5.5 and HCBP-1	Spherical	~30	MRI and FI agent for lung cancer stem cell targeting	[125]
Fe_3_O_4_@SiO_3_-β-CD	FITC and FA	Spherical (core–shell)	70	Magnetic manipulation, bioimaging, cell targeting, and drug delivery	[119]
Fe_3_O_4_@mSiO_2_-PEI	RhB and siRNA	Spherical (yolk-shell)	63	Fluorescence tracking and magnetically guided small interfering RNA delivery	[126]
IONPs-PS	Coumarin-153, IgG, and ICAM-1	Nanowires	7440 × 270	Potential tumor targeting MRI and FI	[127]
Fe_3_O_4_@mSiO_2_	FITC, AlC_4_Pc, and FA	Spherical (yolk-shell)	~50	Tumor targeting MRI, FI, and photodynamic therapy	[120]
Fe_3_O_4_@PAA	Rh123, and FA-PEG	Spherical	~86	Dual-modal molecular imaging	[128]
IONP@SiO_2_-PEG	RhB and cetuximab	Spherical (core–shell)	~60	In vivo colon cancer targeting and imaging	[129]
SPION@SiO_2_	Cy5.5 and RGD	Spherical (core–shell)	~97	Magnetically enhanced cancer imaging and targeting	[130]
DySiO_2_-Fe_3_O_4_	RhB and HmenB1	Spherical	~45	Dual-modal MRI and FI of neuroblastoma	[131]
SPION@dextran	Cy5.5 and EPPT	Spherical	41	Dual-modal in vivo imaging of tumor response to therapy	[132]
MNPs	DiI and anti-EpCAM, or anti-N-cadherin	Spherical	~160	CTC capture, 98.8%	[133]
USPIO/Ag NPs or Au NPs	Anti-MG1	Spheroidal	~47	Photothermal ablation of liver metastases	[134]
IONPs	FITC and anti-EpCAM	Spherical	~200	CTC isolation and detection, 87%	[135]
Fe_3_O_4_@CdSe/ZnS QDs	S. pneumoniae antibodies	Spherical	150	Detection of bacteria	[136]
Fe_3_O_4_@ZIF–8/MoS_2_ QDs	cDNA	-	100	Detection of ATP	[137]
CdTe QDs-Fe_3_O_4_	hCC49 antibodies	Spherical	50	Imaging colon carcinoma cells	[138]
IONP@QDs (*QDs:575, 605, 635, 485*)	anti-HER2 antibodies	-	250 ± 81	Selective cell isolation and counting on smartphone-based imaging platforms	[139]
Fe_3_O_4_@Ag/graphene–QDs	anti-CFP-10 antibodies	Spherical NPs deposited onto glassy carbon electrodes	270	Electrochemical immunosensor for tuberculosis	[140]
Au_rod_-(Fe_3_O_4_)	Herceptin	Nanorods	15	Dual-mode imaging, and photothermal ablation of cancer cells	[121]
Au NC@Fe_3_O_4_	Folic acid	Nanocages	70	Multimodal contrast agents	[141]
Au NS@Fe_3_O_4_ /SiO_2_	Streptavidin	Nanosphere	~100	Magnetic and optical imaging and photothermal therapy	[142]
Fe_3_O_4_@SiO_2_/Ag	Raman-label compounds	Nanosphere (core–shell)	50	Cancer cell targeting	[143]
Fe_3_O_4_@SiO_2_/Ag	Raman-label compounds, and antibody	Nanosphere (core–shell)	50	Cancer cell separation	[143]
IONPs	PPyCOOH, PEG, FA	Irregular	47	AMF triggered MHT and DDS (T_g_) for DOX	[116]
MNPs	PEG-g-p(AAm-co-AN)-A54	Irregular	80	AMF-triggered MHT and DDS (UCST) for DOX	[144]
SPIONs	PEG-(Glu-Hyd)-PEG-FA	Spherical nanovesicles	150	DDS (pH) for DOX, and MRI	[145]
mSiO_2_@ SPIONs	HAMA-b-DBAM-FA	Spherical	~200	DDS (pH) for DOX, and MRI	[146]
Fe_3_O_4_@Gd_2_O_3_	PEG-FA	Yolk-shell	109	DDS (pH) for cisplatin, and dual-mode MRI	[122]
IONPs	poly(*β*-aminoester)-FA	Irregular	50–200	DDS (pH) for DOX, and MRI	[147]
IONPs	mPEG-b-P(DPA-DE)LG	—	~120	pH-triggered MRI probes	[148]
SPIONs	p(HEMA-co-DMA)	Spherical	~20	DDS (pH) for BTZ, and MHT	[149]

**Abbreviations:** IONPs: iron oxide nanoparticles; SPIONs: superparamagnetic iron oxide nanoparticles; MNPs: magnetic nanoparticles; mSiO_2_: mesoporous silica; DPSE-mPEG: 1,2-distearoyl-sn-glycero-3-phosphoethanolamine-N-[methoxy (polyethylene glycol)-2000]; cyclic RGD: cyclo(-Arg-Gly-Asp-D-Phe-Cys) peptide; cyclic RAD: cyclo(-Arg-Ala-Asp-DPhe-Cys) peptide; Cy5.5: cyanine-5.5; gH625: membranotropic peptide gH625; Cy7: cyanine-7; HAS: human serum albumin; DVS: divinyl sulfone; FGF2: fibroblast growth factor 2; p(HEMA): poly(2-hydroxyethyl methacrylate); p(NIPAAM-co-AA): poly(N-isopropylacrylamide-acrylic acid); FITC: fluorescein isothiocyanate; FA: folic acid; PEI: polyethyleneimine; RhB: rhodamine B; HCBP-1: GGLGCF-PEGEMACWWSGGSGK peptide; β-CD: β-cyclodextrin; PS: polystyrene; IgG: immunoglobulin-G; ICAM-1: intercellular adhesion molecule 1 antibody; AlC_4_Pc: tetra-substituted carboxyl aluminum phthalocyanine; PAA: poly(acrylic acid); Rh123: rhodamine 123; FA-PEG: folic acid-linked poly(ethylene glycol); RGD: arginine-glycine-aspartic acid; DySiO_2_: dye-doped silica; HmenB1: HmenB1 antibody; EPPT: C-AHA-A-R-E-P-P-T-R-T-F-A-Y-W-G-K peptide; EpCAM: epithelial cell adhesion molecule; CTC: circulating tumor cell; USPIO: ultrasmall superparamagnetic iron oxide; AMF: alternating magnetic field; MHT: magnetic hyperthermia therapy; MRI: magnetic resonance imaging; DDS: drug delivery system; NIR: near-IR light; LCST: lower critical solution temperature; UCST: upper critical solution temperature;T_g_: glass transition temperature; T_c_: Curie temperature; PLGA: poly(lactic-co-glycolic acid); PNIPAm: poly(N-isopropyl acrylamide); MBAm: *N*,*N*’-methylenebisacrylamide; AAc: acrylic acid; PNVP: poly(1-vinyl-2-pyrrolidone); PEGMEA: polyethylene glycol methyl acrylate); PEG: polyethylene glycol; PPyCOOH: carboxylic polypyrole; PPZ: poly(organophosphazene); P(EO-co-PO)-b-PLL: poly(ethyleneoxide-co-propyleneoxide)-b-poly(L-lysine); PAsp(DIP): poly(2-(diisopropylamino)ethyl aspartate; Glu-Hyd: glutamate hydrozone; mPEG-b-P(DPA-DE)LG: methyloxy-poly(ethylene glycol)-block-poly[dopamine-2-(dibutylamino) ethylamine-L-glutamate]; p(HEMA-co-DMA): poly(2-hydroxyethyl methacrylateco-dopamine methacrylamide); DOX: doxorubicin; BTZ: bortezomib.

## Data Availability

The data presented in this review are available from the original publications that are cited.
